# Serum Protein Panel Facilitates Surgical Decision-Making for Barcelona Stage B Hepatocellular Carcinoma

**DOI:** 10.1016/j.mcpro.2026.101674

**Published:** 2026-10-05

**Authors:** Jin-Hua Wang, Liang-Liang Ren, Wan-Jun Zhang, Xiao-Hua Xing, Jun-Qing Li, Chao-Ying Li, Wei-Jie Qin, Xiao-Long Liu, Ying Jiang

**Affiliations:** 1 State Key Laboratory of Medical Proteomics, National Center for Protein Sciences (Beijing), Beijing, China; 2 Department of Pathology, Institute of Oncology, School of Basic Medical Science, Fujian Medical University, Fuzhou, China; 3 School of Basic Medical Science, Anhui Medical University, Hefei, China; 4 Beijing Proteome Research Center, Beijing, China

**Keywords:** serum proteome, Barcelona Clinic liver cancer B, surgery decision, prognosis, machine learning

## Abstract

To address the clinical heterogeneity that limits uniform treatment recommendations, we developed a serum proteomic-based prognostic model for predicting surgical outcomes in patients with Barcelona Clinic Liver Cancer (BCLC) stage B hepatocellular carcinoma (HCC). Preoperative serum samples from patients with HCC undergoing curative resection were retrospectively collected and profiled using nanoparticle-enhanced data-independent acquisition mass spectrometry. A prognostic risk model was developed in a training cohort (N = 52, BCLC stage B) using 5-fold cross-validated Lasso-Cox machine learning, with overall survival as the primary endpoint. The model was validated in an independent BCLC stage B cohort (N = 22), extended to a single huge HCC cohort (N = 26, BCLC stage A with a solitary tumor ≥10 cm), and benchmarked against a comparison cohort (N = 190, BCLC stage 0/A without huge HCC). Orthogonal validation was performed by parallel reaction monitoring in an exploration cohort (N = 51, BCLC stage B). Among 2381 identified proteins, a three-protein panel (LCP1, DOK3, and CNN2) was selected. The model showed strong predictive accuracy for 3-year overall survival in the training cohort (area under the curve [AUC] = 0.936, sensitivity = 0.988, specificity = 0.834), outperforming the clinical Kinki criteria. It also performed well in the validation cohort (AUC = 0.812, sensitivity = 0.875, specificity = 0.786) and the extended cohort of huge HCC patients (AUC = 0.758, sensitivity = 0.709, specificity = 0.852). Notably, low-risk BCLC stage B patients showed prognosis comparable to the comparison cohort, suggesting potential surgical benefit. Parallel reaction monitoring validation confirmed the model's prognostic value (AUC = 0.800, sensitivity = 0.870, specificity = 0.700). This serum proteomic-based model accurately predicts surgical outcomes for BCLC stage B HCC and outperforms clinical stratification. By identifying low-risk patients who may benefit from surgery, it facilitates personalized treatment planning and may improve survival and quality of life.

## Highlights

• A serum three-protein prognostic model was developed for patients with BCLC stage B HCC using nanoparticles-enhanced DIA-MS and outperformed the Kinki criteria.
• The model demonstrated predictive value for 3-year OS in patients with solitary huge HCC.
• The risk model may support personalized surgical decision-making for patients with BCLC stage B HCC and those with solitary huge HCC.

## Graphical Abstract

**Figure:**
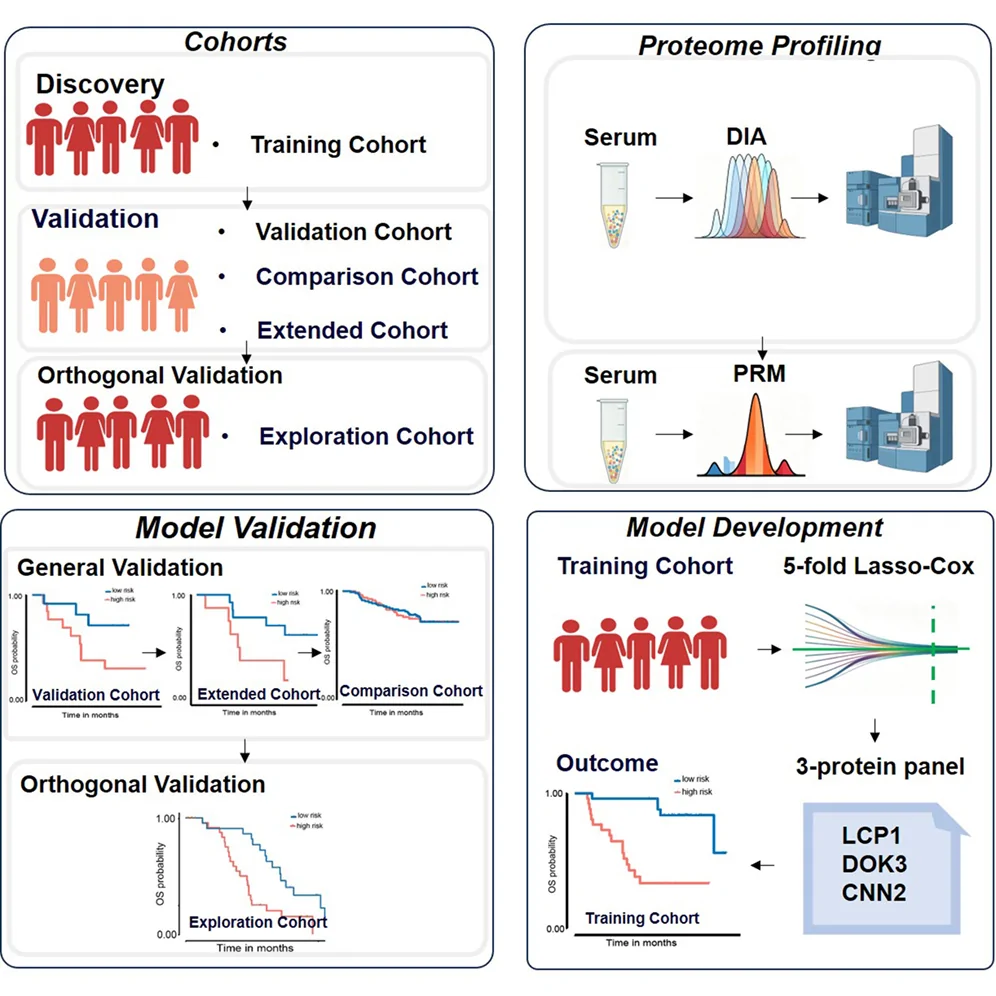

## In Brief

A nanoparticle-enhanced serum proteomic workflow identified a three-protein panel (LCP1, DOK3, and CNN2) that predicts postsurgical survival in BCLC stage B HCC. By identifying low-risk patients with outcomes comparable to BCLC 0/A disease, the model may support individualized selection for curative resection.

Hepatocellular carcinoma (HCC) is the sixth most common malignancy and the third leading cause of cancer death worldwide (1). The Barcelona Clinic Liver Cancer (BCLC) staging system is the most widely used for prognosis prediction and treatment recommendation in patients with HCC (2), which uses the following three variables: physical status (3), liver function of Child-Pugh grade (4), and tumor burden (5) to classify patients into five different disease stages: BCLC stage 0, A, B, C, and D. The criteria for classifying patients with HCC are as follows: BCLC stage 0 is defined as a single nodule ≤2 cm in diameter; stage A is defined as a single nodule >2 cm in diameter, or 2 to 3 nodules ≤3 cm in diameter; the intermediate BCLC stage B HCC is characterized by any one of 2 to 3 nodules >3 cm in diameter, or >3 nodules. The recommended treatments for patients with BCLC stage 0/A HCC include ablation, resection, and liver transplantation. But for patients with a large tumor, especially exceeding 10 cm in diameter, the benefit of surgery remains a controversial issue 6, 7. For stage B patients, resection is not recommended; only transplantation, transarterial chemoembolization (TACE), or systemic treatment (8) are recommended. However, owing to the significant heterogeneity among BCLC stage B HCC patients, the recommended treatment method may not be suitable for all patients in this stage. There are still patients who could achieve a better prognosis through surgical liver resection. In 2013, a study based on a large cohort of HCC patients from Italy and Japan found that a considerable number of BCLC stage B HCC patients underwent surgery and had a better prognosis (9). Furthermore, in 2014, the results of a randomized controlled clinical trial conducted in China demonstrated that BCLC stage B HCC patients who underwent surgery had a significantly better prognosis than TACE-treated patients (10). Therefore, it is particularly important to identify BCLC stage B HCC patients who are likely to benefit from surgical liver resection, facilitating more informed surgical decision-making.

The Bolondi subclassification was the first proposed approach for further subdivision of HCC patients with BCLC stage B (11). It subdivided BCLC stage B into B1-B4 according to the up-to-seven criteria (12) and Child-Pugh grade, and none of the recommended treatments included surgery. In 2015, the recommended treatment for patients in group B1 defined by the Kinki criteria, which were modified from the Bolondi criteria (13), included surgery. However, the Kinki criteria have limited effect on the prognostic stratification of patients with BCLC stage B treated with surgery. The current clinical strategies primarily rely on ultrasonography, computed tomography/magnetic resonance imaging (CT/MRI), and histopathological examinations to facilitate surgical decision-making 14, 15. There is still a lack of reliable molecular markers for prognostic stratification of patients with BCLC stage B HCC.

In addition to clinical imaging, considerable research has been conducted to develop reliable tools for early stratification of the population through integrative multi-omics approaches (16). Serum, as a readily accessible and minimally invasive body fluid, contains a variety of molecular substances produced and exchanged during tissue physiopathological processes, and alpha-fetoprotein (AFP) has been used in the early diagnosis of HCC (17). Moreover, the method we published, which uses surface-functionalized nanoparticles for deep MS-based profiling, can be applied to biomarker discovery (18). To our knowledge, few studies have reported serum biomarkers for prognostic stratification of patients with BCLC stage B HCC following liver resection.

In this study, we combined nanoparticle-based serum proteome enrichment with machine learning algorithms to investigate the stratification of patients with HCC related to prognostic outcomes following liver resection surgery. We developed a prognostic risk score model comprising 3 proteins. Through the risk score assessment, we were able to identify a subpopulation with low risk and favorable prognosis among those undergoing liver resection surgery. Interestingly, the prognostic risk score can also be extended to apply to patients with a single huge HCC (≥10 cm), aiding in the selection of those who may benefit from liver resection surgery. Parallel reaction monitoring (PRM)-based targeted proteomics in an exploration cohort also confirmed the prognostic risk model. This research offers an innovative, less invasive strategy to facilitate surgical decision-making for patients and to screen the appropriate population of patients with BCLC stage B HCC before surgery.

## Experimental Procedures

### Patients and Clinical Data

The retrospective study was approved by the ethics committee of the Mengchao Hepatobiliary Hospital of Fujian (Approval No. 2019-066-01, dated December 27, 2019), and all patients provided written informed consent. All procedures were performed in accordance with the ethical standards of the institutional research committee and with the 1964 Helsinki Declaration and its later amendments. We collected preoperative serum samples from patients with histopathologically confirmed hepatocellular carcinoma at stage 0, A, or B according to the Barcelona Clinic Liver Cancer (BCLC) staging system (19) (Supplemental Table S1), who underwent curative liver resection at the Mengchao Hepatobiliary Hospital of Fujian Medical University between March 2013 and February 2017 and had no prior history of preoperative anticancer treatment. Based on the criteria of histopathological confirmation and quality control (QC) assessment 20, 21 with proteomic data (Supplemental Fig. S1), 290 HCC patients (255 males and 35 females, 22–82 years old) were recruited, including 74 at BCLC stage B and 216 at BCLC stages 0/A, with a median overall survival (OS) of 48.50 months (Supplemental Tables S1 and S2). The demographic and clinicopathological information of the patients, such as gender, age, tumor number, tumor size, MVI, lymphatic metastasis, BCLC stage, TNM stage, AFP (ng/ml), ALT (u/l), AST (u/l), TBIL (μmol/l), HBeAg, HBV-DNA, antiviral therapy, HBV, HCV, liver cirrhosis, differentiation, cancer recurrence, DFS (month), death due to recurrence and OS (month), were presented in Supplemental Table S1. In addition, an independent PRM validation cohort (N = 51; 44 males and 7 females, 31–75 years old; ethical approval No. 2019-066-01, dated December 27, 2019), comprising BCLC stage B patients with serum samples previously collected from the hospital biobank, was specifically included for targeted verification at the protein level to provide orthogonal validation. The clinical characteristics of the orthogonal validation of exploration cohort are summarized in Supplemental Table S3.

The standard operating procedures (SOPs) for serum collection were performed following the same protocols as described in the published research (22). Briefly, serum samples were collected using plain collection tubes without anticoagulant. The blood was allowed to clot at room temperature for 30 min and then centrifuged at 1000*g* for 10 min. The supernatant serum was collected and stored at −80 °C until further use. All samples underwent fewer than three freeze–thaw cycles.

### Protection of Human Subject Confidentiality

To protect patient confidentiality, the following mechanisms were employed: immediately after collection, each serum sample, and its accompanying clinical data were de-identified. Any direct identifiers (patient name, medical record number, ID card number) were removed and replaced with a unique study code (format: PL-XXX). The mapping between study codes and patient identities is stored in an encrypted file on a password-protected system, and access to this mapping is held only by the principal investigator and the designated data manager. All downstream experimental processing (nanoparticle enrichment, DIA-MS), and data analysis were performed solely using the coded samples. No patient identifiers appear in any figures, tables, or supplementary files.

### Cohort Description

A total of four main independent cohorts were assigned in this study, including the training cohort, validation cohort, extended cohort, and comparison cohort, which were all derived from the Mengchao Hepatobiliary Hospital of Fujian Medical University, with serum samples collected between March 2013 and February 2017. All cohorts were processed in the same batch using nanoparticle enrichment followed by data-independent acquisition mass spectrometry (DIA-MS) to ensure technical consistency.

The training cohort (N = 52, Table 1) was randomly split from the original 74-patient set with histopathologically confirmed hepatocellular carcinoma (BCLC stage B) and underwent curative liver resection without prior anticancer treatment.

**Table 1:** Clinicopathological characteristics of patients with BCLC stage B HCC

Characteristics	Total BCLC stage B HCC patients	Training cohort	Validation cohort	*p* value
	(N = 74)	(N = 52)	(N = 22)	
Baseline information				
Gender				0.259
Female	8 (10.8%)	7 (13.5%)	1 (4.5%)	
Male	66 (89.2%)	45 (86.5%)	21 (95.5%)	
Age, year				0.171
≤60	52 (70.3%)	39 (75.0%)	13 (59.1%)	
>60	22 (29.7%)	13 (25.0%)	9 (40.9%)	
Laboratory test				
AFP (ng/ml)				0.120
≤400	50 (67.6%)	38 (73.1%)	12 (54.5%)	
>400	24 (32.4%)	14 (26.9%)	10 (45.5%)	
ALT (u/l)				0.313
<50	51 (68.9%)	34 (65.4%)	17 (77.3%)	
≥50	23 (31.1%)	18 (34.6%)	5 (22.7%)	
AST (u/l)				0.880
<40	38 (51.4%)	27 (51.9%)	11 (50.0%)	
≥40	36 (48.6%)	25 (48.1%)	11 (50.0%)	
TBIL (μl/mol)				0.724
<17.1	31 (41.9%)	19 (36.5%)	12 (54.5%)	
≥17.1	43 (58.1%)	33 (44.6%)	10 (45.5%)	
HBV DNA copy number				
<2000	38 (51.4%)	27 ((51.9%))	11 (50.0%)	0.880
≥2000	36 (48.6%)	25 (48.1%)	11 (50.0%)	
Liver function				
Liver cirrhosis				0.105
absence	11 (14.9%)	10 (19.2%)	1 (4.5%)	
present	63 (85.1%)	42 (80.8%)	21 (95.5%)	
Tumor characteristics				
TNM stage				0.197
II	32 (43.2%)	25 (48.1%)	7 (31.8%)	
IIIA	42 (56.8%)	27 (51.9%)	15 (68.2%)	
Diameter of tumor (cm)				0.602
≤10	63 (85.1%)	45 (86.5%)	18 (81.8%)	
>10	11 (14.9%)	7 (13.5%)	4 (18.2%)	
Tumor Number				0.356
2	15 (25.3%)	12 (23.1%)	3 (13.6%)	
≥3	59 (79.7%)	40 (76.9%)	19 (86.4%)	
Microvascular Invasion				0.357
absence	26 (35.1%)	20 (38.5%)	6 (27.3%)	
presence	48 (64.9%)	32 (61.5%)	16 (72.7%)	
Differentiation				0.483
Edmondson I-II	21 (28.4%)	16 (30.8%)	5 (22.7%)	
Edmondson III-IV	53 (71.6%)	36 (69.2%)	17 (77.3%)	

BCLC, Barcelona Clinic Liver Cancer; HCC, hepatocellular carcinoma.

The validation cohort (N = 22, Table 1) comprised the remaining patients from the 74-patient BCLC stage B set, with no significant bias in clinicopathological characteristics with the training cohort, as confirmed by chi-square testing.

To evaluate the generalizability of our proteomic signature, the extended cohort (N = 26) was selected, comprising patients with a single huge hepatocellular carcinoma (histopathologically confirmed HCC, BCLC stages A, diameter ≥10 cm) who underwent curative liver resection without prior anticancer treatment. Clinical characteristics of this cohort are summarized in Supplemental Tables S1 and S2.

The comparison cohort (N = 190) comprised the remaining BCLC stage 0/A patients, and the clinical characteristics of this cohort are summarized in Supplemental Tables S1 and S2.

In addition, an independent PRM validation cohort (N = 51), comprising BCLC stage B patients with serum samples previously collected from the hospital biobank, was specifically included for PRM-based targeted verification for orthogonal validation. The clinical characteristics of this cohort are summarized in Supplemental Table S3.

### Synthesis of Modified SiO_2_ and TiO_2_ Nanoparticles

Modified SiO_2_ and TiO_2_ nanoparticles were synthesized according to previously reported methods 18, 23, with minor modifications. Hydroxyl-modified SiO_2_ nanoparticles were prepared using a modified Stöber method, in which tetraethyl orthosilicate (TEOS) was used as the silica precursor and reacted in an ethanol/water/ammonia mixture at room temperature for 2 h. Amino-modified SiO_2_ nanoparticles were obtained by reacting the hydroxyl-modified particles with (3-aminopropyl) triethoxysilane (APTES) in ethanol for 24 h, followed by washing and freeze-drying. TiO_2_ nanoparticles were synthesized using a solvothermal method. Briefly, TiCl_4_ was neutralized with ammonia to pH 8.0, and the resulting precipitate was hydrothermally treated in ethanol at 150 °C for 10 h. After grinding and sieving, TiO_2_ particles with sizes ranging from 200 to 500 nm were obtained. For the preparation of zwitterion-modified SiO_2_ nanoparticles, a surface-initiated atom transfer radical polymerization (SI-ATRP) initiator was first immobilized onto hydroxyl-modified SiO_2_ nanoparticles at 37 °C for 12 h. Surface polymerization was subsequently conducted with the zwitterionic monomer N,N-dimethyl-N-(acryloyloxyethyl)-N-(3-sulfopropyl) ammonium chloride in toluene for 4 h. The resulting particles were vacuum-dried to obtain a white powder with a particle size of 200 to 500 nm.

Following synthesis, equal masses of hydroxyl-modified SiO_2_, amino-modified SiO_2_, zwitterion-modified SiO_2_, and TiO_2_ nanoparticles were combined. The mixed nanoparticles were dispersed in deionized water at a total concentration of 10 mg/ml to form a homogeneous suspension. Further synthesis details are provided in the Supplementary Information.

### Serum Sample Preparation

Before use, 10 μl of the mixed nanoparticle suspension, corresponding to 100 μg of nanoparticles, was subjected to buffer exchange into binding buffer containing 10 mM Tris-HCl (pH 7.4), 150 mM KCl, and 0.05% CHAPS. The buffer-exchanged nanoparticles were added to 200 μl of serum and incubated at 37 °C for 1 h. Following incubation, the mixture was centrifuged at 20,000*g* for 60 min at 4 °C. The supernatant was carefully removed, and the nanoparticle pellet was washed with 600 μl of binding buffer containing 10 mM Tris-HCl (pH 7.4), 150 mM KCl, and 0.05% CHAPS. The sample was centrifuged again at 20000*g* for 60 min at 4 °C, and the washing solution was discarded. The washed pellet was resuspended in 20 μl of lysis buffer containing 0.5% SDC, 10 mM TCEP, 40 mM CAA, and 50 mM TEAB (pH 8.5), followed by sonication for 10 min. The sample was heated at 95 °C for 10 min and subsequently diluted with 80 μl of 50 mM TEAB to reduce the final SDC concentration to 0.1%. Proteins were digested using a two-step trypsinization procedure, consisting of an initial incubation at 37 °C for 4 h followed by an additional incubation at 37 °C for 12 h. After digestion, 20% TFA was added to achieve a final TFA concentration of 1%. The acidified sample was incubated in an ice bath for 10 min and centrifuged at 15,700*g* for 10 min. The resulting pellet was washed twice with 0.5% TFA. The peptide-containing supernatants were combined and dried at 60 °C before LC-MS/MS analysis.

### Serum Peptide Preparation for Data-Dependent Acquisition (DDA) Analysis

The pooled peptide samples used for DDA analysis were derived from 10 patients distributed across BCLC stages 0/A/B from 290 patients, and then were separated using a small reversed-phase high-performance liquid chromatography column (sRP-HPLC). In summary, approximately 1 μg of peptide from each sample was combined, and the pooled peptide solution was eluted in a gradient of 0%, 5%, 10%, 15%, 20%, 25%, and 40% ACN concentration in ammonia with pH 10 spanning 45 min. Subsequently, the fractions eluted at 0% and 40% ACN concentrations were combined. Ultimately, a total of six fractions were obtained and stored at −80 °C for DDA analysis, and each fraction was measured in duplicate (technical replicates) to construct the serum spectral library.

### DDA Analysis

The serum peptide samples were analyzed on an EASY-nLC 1200 liquid chromatography system (Thermo Fisher Scientific) coupled with a Q Exactive HF mass spectrometer (Thermo Fisher Scientific). Initially, the peptides were separated on a trapping column (3 μm, 120 Å, C18 AQ resin, 20 mm × 100 μm i.d., from Dr Maisch GmbH Inc.) and were eluted over an analytical column (1.9 μm, 120 Å, C18 AQ resin, 300 mm × 150 μm i.d.) at a flow rate of 600 nl/min. Mobile phase A consisted of 0.1% formic acid, while mobile phase B was a mixture of acetonitrile and 0.1% formic acid. The elution gradients for peptide separation were as follows: 0 to 8 min, 7 to 12% B; 8 to 70 min, 12 to 30% B; 70 to 82 min, 30 to 45% B; 82 to 85 min, 45 to 95% B; and 85 to 90 min, 95% B.

For DDA, MS data were acquired using a Top 25 data-dependent MS/MS scan method (top N method) in positive ion mode. The full MS spectra scan was obtained using the Orbitrap, with a maximum injection time of 80 ms and an m/z resolution of 120,000 at 200 m/z, within the range of 400-1200 m/z. The secondary MS/MS scans were performed at a resolution of 15,000 at m/z 200, with a fixed first mass of 120 m/z and a maximum injection time of 45 ms. Fragmentation of precursor ions was achieved through higher-energy C-trap dissociation (HCD) with a normalized collision energy of 27%. To prevent repeated sequencing of identical peptides, a dynamic exclusion duration was set to 18 s.

### Spectral Library Generation Constructed by MaxQuant-Based Database Identification Using DDA Data

All DDA MS data, originating from a total of six fractions each analyzed twice in DDA mode, were searched against the UniProt Human database (uniprot_Homo_sapiens_20220217_20375.fasta) using the MaxQuant search engine (version 2.0.3.0). The parameters for database searching in MaxQuant were as follows: trypsin was chosen as the proteolytic enzyme, permitting up to 2 missed cleavages; carbamidomethyl (C) was set as a fixed modification, while acetyl (protein N-term) and oxidation (M) were selected as variable modifications. Mass tolerances were as follows: for precursor ions, a first search tolerance of 20 ppm (for mass recalibration) and a main search tolerance of 4.5 ppm; for fragment ions, the mass tolerance was 0.02 Da. The search results were filtered at both the protein and peptide levels, ensuring a 1% false discovery rate (FDR). The minimum allowed peptide length was set to 7 amino acids, and the maximum peptide mass was limited to 4600 Da. Following the MaxQuant database search, the resulting “combined/txt” folder (containing evidence.txt, msms.txt, and associated files) was used as input for Spectronaut spectral library construction. All peptides identified in the DDA data analysis, including the following information: peptide sequence, precursor mass and mass/charge, observed modifications (*e.g*., oxidation, acetylation, etc.), and identification scores are listed in the Supplemental Table S4.

### Data-Independent Acquisition (DIA) Data Search Using Spectronaut

The DDA-based spectral library was generated by importing the “evidence.txt” file from a MaxQuant search (default parameters, FDR <1% at PSM and peptide levels) into Spectronaut (version 14.10.201222) *via* the “Library Generation” module using the “Import MaxQuant results” option. The resulting library was saved in the proprietary ∗.kit format, which contains precursor m/z, fragment ion m/z, relative fragment intensities, and normalized retention times (iRT). No additional PTM localization or in silico library optimization was performed at this stage.

The DIA raw files were imported into Spectronaut and analyzed using the “BGS Factory Settings” default workflow with the following key parameters:

(i) XIC extraction & mass tolerance: Both MS1 and MS2 mass tolerances were set to Dynamic (correction factor = 1). Retention time (RT) extraction windows were also dynamic (correction factor = 1).
(ii) Calibration: Automatic calibration was applied using a local (nonlinear) iRT-to-RT regression. The Biognosys iRT kit was used for RT standardization, and deamidated peptides were excluded from calibration.
(iii) Identification & FDR control: A decoy library was generated using the Mutated method. Machine learning was performed per run. Precursor and protein q-values were both controlled at < 0.01 (1% FDR).
(iv) Exclusion of single-hit proteins: The option “Exclude Single Hit Proteins” was set to True; consequently, proteins identified by only one unique peptide were removed from the final protein list.
(v) Quantification: Quantification was performed at the MS1 level using peak area, with all other acquisition and processing parameters set to default. The results of the proteomic data were shown in Supplemental Table S5.

### DIA Scheme

Serum proteomic profiling was performed using a protein quantitation platform integrating nanoparticles protein coronas with data-independent acquisition (DIA)-mass spectrometry approach 24, 25, 26, 27, 28. A 1-μg aliquot from each sample was measured using an LC-MS instrument, specifically an EASY nLC 1200 ultra-high-pressure system (Thermo Fisher Scientific) coupled with a nano-electrospray ion source to a Q-Exactive HF (Thermo Fisher Scientific). The column size and %B gradient were identical to those used for DDA. For DIA measurements, the scanning mode was positive ion scanning mode, the primary mass spectrum was detected by Orbitrap with a resolution of 60,000, the maximum injection time was 80 ms, and the scanning range was 400-1200 m/z, the scan resolution was set to 30,000, the starting scan mass was 200 m/z, and the maximum ion injection time was 45 ms; the isolation window for each acquisition was 12 m/z, the number of isolation windows was 41, and the normalized collision energy of HCD was 27%.

### QC of DIA MS Data

The QC of serum proteomic data comprised three sections: peptide preparation, stability of mass spectrometer monitoring, and MS raw data cleaning. The monitoring of peptide preparation and stability of mass spectrometer contains 293T cell qualification, pooled sample preparation, QC qualification, and spike-in proteins of indexed retention time (iRT, Biognosys). For the peptide preparation, the order of sample preparation for all samples was randomly distributed, and a QC sample was prepared for each batch. To ensure the stability of the mass spectrometer, a QC sample was run every 20 samples, and 293T cell samples were performed daily. Furthermore, a mixture of 11 standard peptide samples, known as iRT peptides, was spiked into each sample before MS injection to align the data and assess the quantitative accuracy of the chromatographic mass spectrometry system. The number of iRT peptides exceeded 8, and the retention time of each iRT peptide in serum samples was controlled to within ±5 min across the cohort. Pearson’s correlations of protein quantification between QC samples from different batches were calculated to estimate the technical process stability throughout the data output (Supplemental Fig. S1*A*).

### The Method of Grouping Patients

The randomly selected 52 patients (7/10 of the total BCLC stage B patients) were assigned to a training cohort, while the remaining 22 patients (3/10 of the total BCLC stage B patients) were assigned to the validation cohort. The absence of grouping bias in clinicopathological characteristics between the training cohort and the validation cohort was confirmed by the chi-square test.

### Model Construction of Prognostic Biomarker Panel

Initially, univariate Cox regression for overall survival (OS) with a significance level of *p* < 0.05 was employed to identify prognosis-related proteins. Subsequently, four proteins were randomly selected from the prognosis-related proteins to form a panel. This process was repeated 15,000 times, resulting in a total of 12,203 candidate biomarker panels. The expression level of each protein in all samples was normalized using the z-score method, and a 5-fold cross-validation Lasso-Cox regression analysis was applied to each panel. Within each fold, the data were randomly divided into a training set and a testing set, maintaining a 4:1 ratio. Proteins with a non-zero weight in the panel were retained, and if the number of remaining proteins in a panel was ≤1, then the panel was discarded. The coefficient for each protein in the training set was employed in the testing set, and the risk score for each patient in the training and testing sets was calculated as the sum of the normalized expression of each protein multiplied by its corresponding coefficient.

The formula of the risk score was as follows: RiskScore=coefficient1∗protein1)+(coefficient2∗protein2)+...+(coefficientn∗proteinn). A 5-fold cross-validation was performed on the entire panels. The risk score for each patient was calculated, and its predictive performance for 3-years OS in patients with HCC was assessed using the area under the curve (AUC). Panels with a mean AUC for OS greater than 0.85 at 3 years in both the training set and the testing set were selected for the next step of candidate panel optimization. For those selected panels, we conducted 100 iterations of 5-fold cross-validation to refine the results. The panel achieving the highest mean AUC in the testing set at 3 years was identified as the final optimal prognostic biomarker panel. Finally, the hazard ratio (HR) for each protein in this optimal panel was estimated using univariate and multivariate Cox regression analysis of OS.

The formula for calculating the patients' prognosis risk score is as follows:

(1) RiskScore=LCP1∗0.338+DOK3∗(−0.427)+CNN2∗(−0.150)

### Model Prediction in the Discovery, Validation, and Orthogonal Validation Step

The transformed log2 intensities for the training cohort, the validation cohort, the extended cohort, the comparison cohort and the exploration cohort were normalized using z-score, separately. The median risk score of patients in the training cohort was used as the cut-off value to divide each cohort into low-risk and high-risk groups. Kaplan-Meier (K-M, log-rank test) survival analysis was implemented to distinguish the difference in OS between the low-risk and high-risk groups, with a *p* value cut-off of 0.05 for the log-rank test.

### Comparison of the Predictive Ability of the Kinki Criteria and Our Prognostic Risk Model

The comparison between the clinical grouping method of the Kinki criteria (12) and our prognostic risk model was conducted in the BCLC stage B HCC cohort. The patients were grouped according to their respective standards: the Kinki criteria relied on clinical categories, whereas our prognostic risk model utilized risk scores. For the Kinki criteria method, patients with BCLC stage B HCC were categorized into B1 and non-B1 groups, which aligned with the low-risk and high-risk groups. For our prognostic risk model, patients with BCLC stage B HCC were classified into low-risk and high-risk groups based on the median risk score value derived from the BCLC stage B HCC training set. We then combined the clinical information on OS to assess the stratification performance of the two methods using a log-rank test.

### DIA Quantification

The protein identification was performed on Spectronaut (version 14.10.201222) using the constructed data-dependent acquisition (DDA) spectral library and the UniProt Human database (version 2021. 11.05), which was used as the search database and is available at https://www.uniprot.org/. The parameters were set to “BGS Factory Settings” (default), with “MS1” selected for Quantity MS-Level, “Exclude Single Hit Proteins enabled,” and other parameters left were set by default.

### The Quality-Control Statistical Method for MS Data

Pearson's correlation coefficient was calculated for all QC runs in the statistical analysis environment R (v4.1.3). The raw proteome data of serum samples were log2-transformed using R (version 4.2.2), and outliers smaller than QL - 2 × IQR were replaced with NA. QC consisted of three main aspects: (*i*) samples with abnormal numbers of identified proteins, peptides, or precursors exceeding QU + 1.5 × IQR were removed (Supplemental Fig. S1*B*); (*ii*) the total percentage of erythrocyte contamination protein markers was calculated according to the existing literature (20), and samples with values exceeding two standard deviations from the mean, together with known hemolytic samples, were considered as red blood cell-contaminated samples (Supplemental Fig. S1*C*), the list of protein contaminants in the QC serum was shown in Supplemental Table S6; (*iii*) robust principal component analysis (PCA) was performed using the robPCA function in R (version 4.2.2) to remove outlier samples (21) (Supplemental Fig. S1*D*). As a preliminary QC step, proteins with valid values in less than 70% of all samples were excluded to remove undetectable noise. Subsequently, to prevent data leakage, the samples were strictly divided into the training, validation, and extended cohorts before any further numerical preprocessing. Quantile normalization, global minimum imputation, and scaling were then performed independently within each cohort. For risk score calculation, the protein coefficients and cut-off value derived from the training cohort were applied to the validation cohorts for outcome prediction.

### Development and Analytical Validation Targeted MS Measurements

According to the MCP targeted MS guidelines (29), the targeted assay described here corresponds to a Tier 3 measurement. This classification is appropriate because our study aimed at relative comparisons of candidate protein panels across sample groups in an early-stage discovery context. To construct the PRM spectral library, DDA was performed three times on pooled serum samples using an Orbitrap Exploris 480 mass spectrometer equipped with a FAIMS Pro interface. Samples were loaded onto a trap column (100 μm × 2 cm) at 2.5 μl/min, followed by separation on an analytical column (home-made, 75 μm × 20 cm) at 300 nl/min. The gradient of mobile phase (0.1% formic acid in water) was as follows: 0 min, 5% B; 0 to 5 min, 5%–12% B; 5 to 53 min, 12%–30% B; 53 to 63 min, 30%–45% B; 63 to 69 min 45%–95% B; and 69 to 78 min, 95% B. For DDA, the FAIMS Pro interface was operated with two compensation voltages (CVs) of −45 V and −65 V. MS was performed with a resolution of 60,000 (at 200 m/z) and an m/z range of 350-1,500, using an electrospray voltage of 2200 V. Target ions underwent MS/MS using HCD, with a resolution of 17,500 (at 200 m/z) and a 1.6 m/z isolation window. The DDA data were searched against the target protein sequence database using MaxQuant (version 2.8.0), and the resulting identifications-including precursor masses, charge states, fragment ion spectra, and retention times of the selected peptides-were compiled into the library for scheduling PRM acquisition.

For PRM-based quantification of the target proteins comprising the prognostic panel, serum from the exploration cohort was analyzed. For each target protein, candidate peptides were initially filtered from a DDA library using criteria including uniqueness, sequence length (6-30 aa), complete trypsin cleavage, allowance of at most one missed cleavage, and exclusion of labile residues. Following PRM validation in real samples, 3 to 6 fragment ions with optimal signal-to-noise and minimal interference were chosen per peptide. The summed peak areas of these ions (exported as 'Total Area' from Skyline) were used for final quantification. PRM analysis was performed on the same Orbitrap Exploris 480 mass spectrometer and with the same liquid chromatography gradient program as used for DDA acquisition. The instrument was operated in positive ion mode, with a default charge state of 3. For PRM, FAIMS was operated with the same two compensation voltages (CVs) of −45 V and −65 V. Full MS precursor scans were acquired over a mass range of m/z 350-1500 at an Orbitrap resolution of 60000 (m/z 200), with an AGC target of 3 × 10^6^ and automatic maximum injection time, while targeted MS/MS for the selected peptides was performed in scheduled PRM mode within ±5 min retention time window around their DDA-predicted retention times. Targeted precursor ions were isolated in the quadrupole with an isolation window of 1.6 m/z and fragmented by HCD at a normalized collision energy of 27%. MS/MS spectra were acquired in the Orbitrap at a resolution of 15000 (m/z 200), with an AGC target set to standard and automatic maximum injection time. The first mass was fixed at m/z 120.

For quality control, PRM QC samples were prepared by combining equal aliquots of tryptic peptides from an orthogonal validation cohort and then interspersed among the analytical runs. The following metrics, namely retention time (within ±2 min), peak shape deviation (Supplemental Fig. S2*A*), and quantitative repeatability (Pearson correlation coefficient; see Supplemental Fig. S2*B*), were assessed. These measures confirm the stability and reproducibility of our Tier 3 PRM measurements.

PRM data were analyzed using Skyline (version 26.1.0.057). For each peptide, the peak area was calculated as the sum of peak areas of at least five transition ions. Following extensive empirical comparison of chromatographic behavior, ionization efficiency, and fragmentation patterns across repeated injections, one unique peptide per candidate protein that met these criteria was ultimately selected for targeted validation using PRM-MS. The list of peptides analyzed in the PRM experiments is detailed in Supplemental Table S7.

### Experimental Design and Statistical Rationale

A total of 290 HCC serum samples were analyzed as biological replicates, along with QC samples injected every 22 runs for instrument monitoring (Pearson's r > 0.9 for QCs, Supplemental Fig. S1*A*). Instead of conventional power analysis typically used for differential expression, we established statistical significance through extensive internal validation: 15,000 random protein panels selections, 5-fold cross-validation (4:1 split) repeated 100 times, and a permutation test (1000 iterations) confirming that the final AUC >0.85 is highly unlikely by chance (*p* < 0.001), ensuring robust and generalizable prognostic predictions. The selected candidate panel was validated by targeted PRM on an Exploris FAIMS 480 mass spectrometer. PRM QC samples from an orthogonal validation cohort were interspersed among the analytical runs, and we monitored retention time deviation (within ±2 min) and quantitative reproducibility (Pearson's r > 0.85).

### Statistical Analysis

Quantification and statistical analysis methods for omics analyses were mainly presented and cited in the STAR Methods details provided in Supplemental Table S8. The time-dependent receiver operating characteristic curve (ROC) was plotted by the “survival ROC” package with a cut-off time of 3 years. The “survival” package was used to analyze the proteins in the panel to assess the dependence of risk factors. K-M survival analysis was implemented using the “Survminer” package.

In the BCLC stage B HCC training cohort, the risk score and other clinical baseline characteristics, such as gender, age, AFP, ALT, AST, TBIL, HBV DNA copy number, liver cirrhosis, and differentiation, were analyzed by univariate and multivariate Cox regression analysis using a significance level of *p* < 0.05 to calculate the HR. Among them, risk score, and age, were continuous variables, while others were categorical variables.

## Results

### Patients’ Characteristics

Serum samples were initially obtained from 312 HCC patients (BCLC stages 0/A/B) for DIA proteome. After proteome data quality filtering, 22 samples with abnormal contamination were excluded, and 290 samples were finally retained, including 74 BCLC-stage B and 216 BCLC-stage 0/A HCC patients (Supplemental Fig. S1*E* and Supplemental Table S1). The detailed workflow of the study was shown in Figure 1. A total of 74 patients with BCLC stage B were randomly assigned into the training cohort (N = 52, 7/10 of the total) and the validation cohort (N = 22, 3/10 of the total), with no significant bias in clinicopathological characteristics as confirmed by the chi-square test (Table 1). Additionally, 26 patients with a single huge HCC were designated as the extended cohort. The remaining 190 BCLC stage 0/A patients were designated as the comparison cohort. The clinical baseline features of the comparison and extended cohorts are shown in Supplemental Tables S1 and S2. An independent cohort with 51 BCLC stage B HCC patients was used for orthogonal validation by PRM-targeted proteome to confirm the feasibility of the prognostic model. Baseline data for this exploration cohort are shown in Supplemental Table S3.

**Fig. 1:**
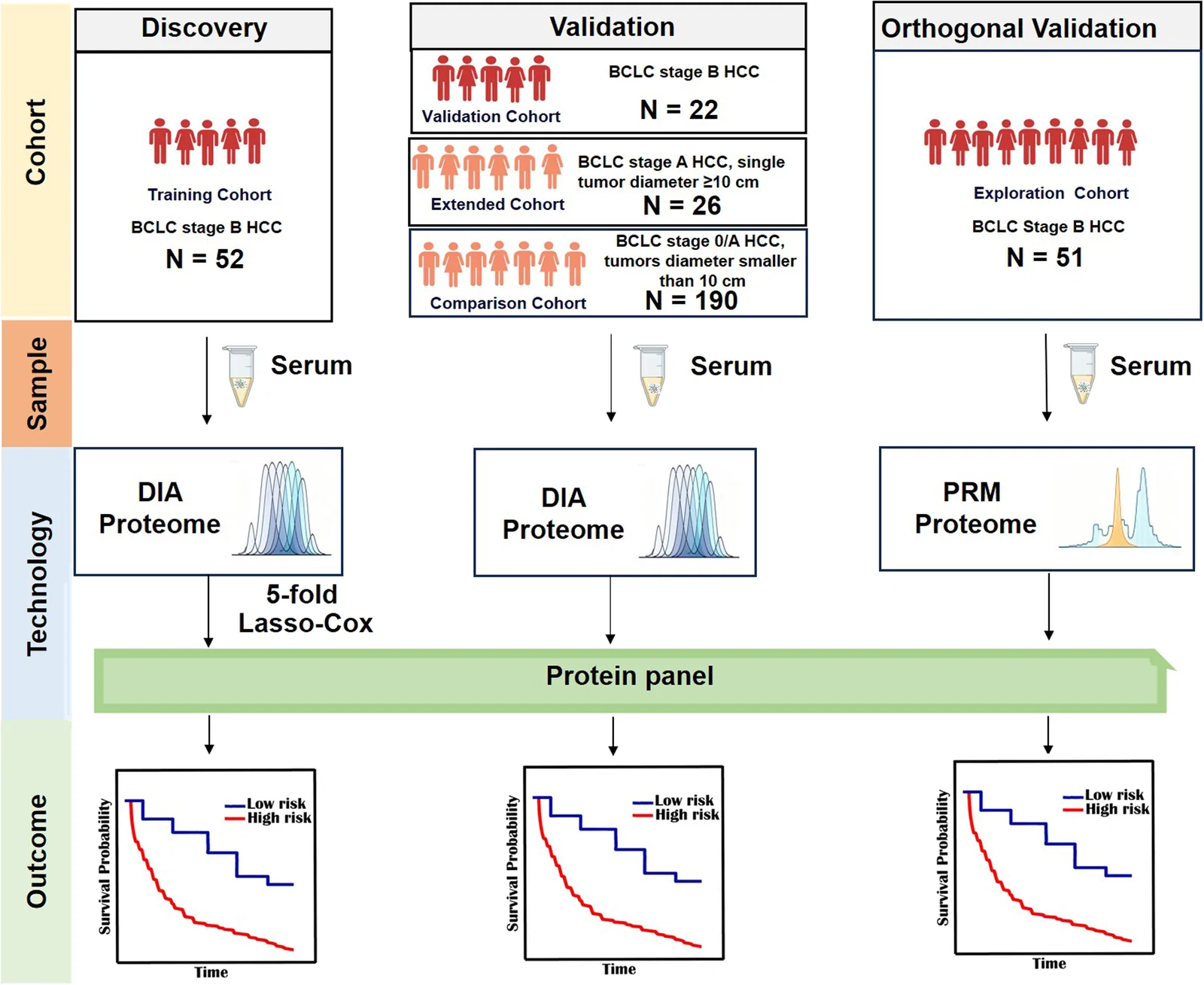
**Study workflow for developing and verifying a serum-protein prognostic signature for HCC.** In the discovery cohort (52 BCLC B HCC patients), DIA together with fivefold Lasso-Cox analysis was used to establish a protein panel. DIA-based validation was conducted in three independent cohorts (N = 22, 26 and 190 respectively). An independent exploration cohort (51 BCLC B HCC patients) was tested by PRM for orthogonal validation. Kaplan–Meier survival analysis was applied in each cohort to separate high-risk and low-risk groups and assess survival outcomes. BCLC, Barcelona Clinic Liver Cancer; Cox, Cox proportional hazards model; DIA, data-independent acquisition; HCC, hepatocellular carcinoma; Lasso, Least Absolute Shrinkage and Selection Operator; PRM, parallel reaction monitoring.

### Serum Proteomic Profiling of Patients With HCC

With nanoparticle enrichment, following a strict QC assessment of proteomic data 20, 21 (Supplemental Fig. S1), a total of 2378 proteins were identified from 290 HCC serum samples (Supplemental Table S1). The distribution of identified protein numbers per sample is shown in Figure 2*A*, with a mean of 1426 proteins and a range of 666-2057(Fig. 2*A* and Supplemental Figs. S3 and S4). It can be seen that the identified serum proteome spanned six orders of magnitude in abundance (Fig. 2*B*), reflecting the broad dynamic range inherent to serum samples. Known or potential HCC biomarkers, including CCL5, AFP, CHI3L1, GDF15, TGFB1, SPP1, MDK 17, 30, and so on, are annotated on the ranked plot in Figure 2*B* with distinct labels. This high-quality proteomic dataset provided a solid foundation for subsequent prognostic model development and validation using DIA -based approaches.

**Fig. 2:**
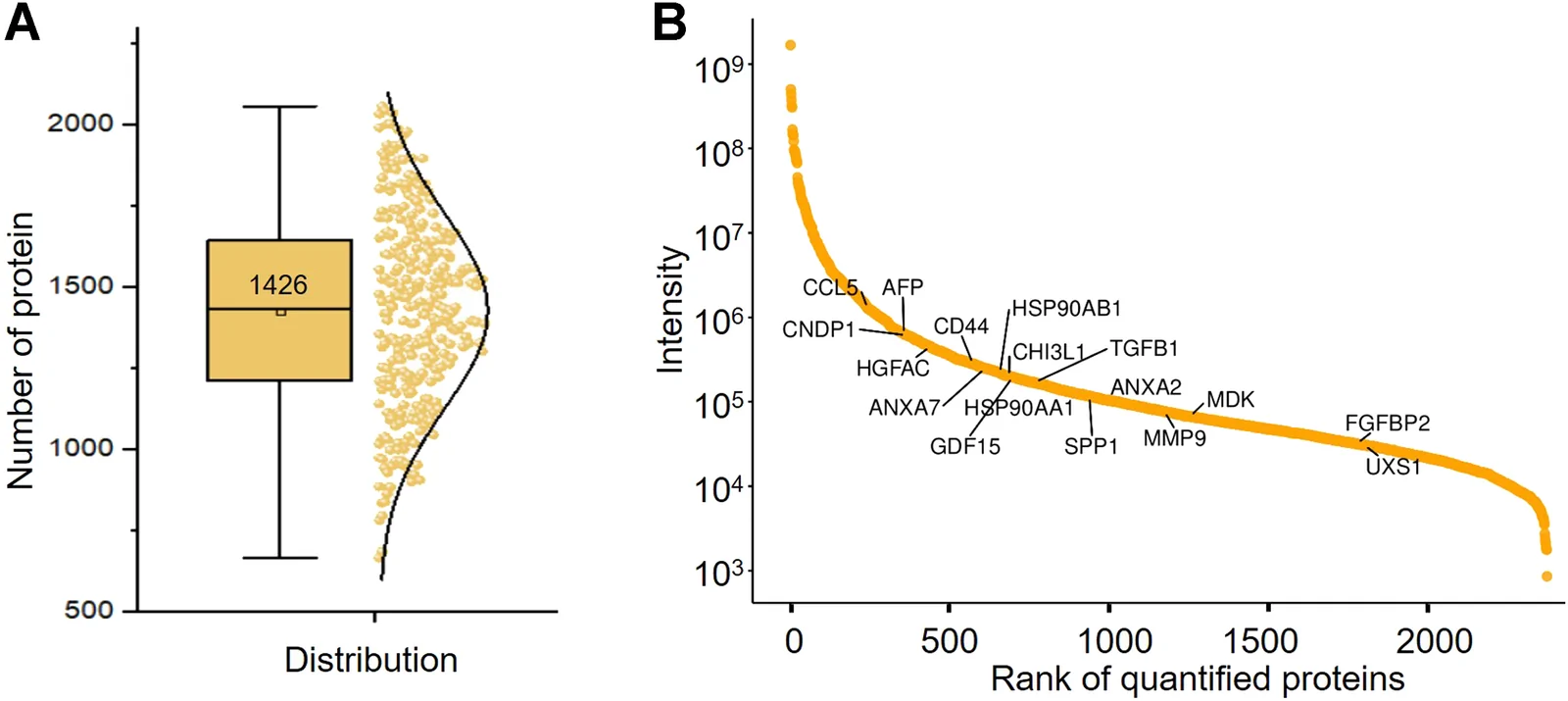
**The output of MS-based serum proteome profiling.***A*, the distribution of protein number identified from 290 HCC serum samples. *B*, identified proteins are ranked by mean intensity. Known and potential HCC biomarkers are highlighted with text labels along the intensity curve. Source data are provided as in Supplemental Table S5. HCC, hepatocellular carcinoma; MS, mass spectrometry.

### Machine-Learning-Based Selection of Biomarker Combinations for Classification of BCLC Stage B HCC Patients

We acquired 121 proteins by integrating univariate Cox screening, which were significantly related with the prognosis outcomes of BCLC stage B HCC patients. Aiming at establishing a reliable and accurate potential prognostic prediction model based on the serum proteomic data, we constructed a pipeline by adopting the BCLC stage B HCC training cohort and stepwise Lasso-Cox regression machine learning algorithm parameter optimization for analysis (Fig. 3*A*), subsequently got a compact biomarker combination containing three hub proteins to construct the prognostic risk score, including lymphocyte cytosolic protein 1 (LCP1), docking protein 3 (DOK3), and calponin-2 (CNN2). The pipeline contains three steps, including the selection of significantly prognosis-associated, highly ranked candidate proteins, the generation of 12203 groups from 15000 initial protein combinations, and final biomarker determination using multi-step 5-fold Lasso Cox cross-validation (Fig. 3*A*).

**Fig. 3:**
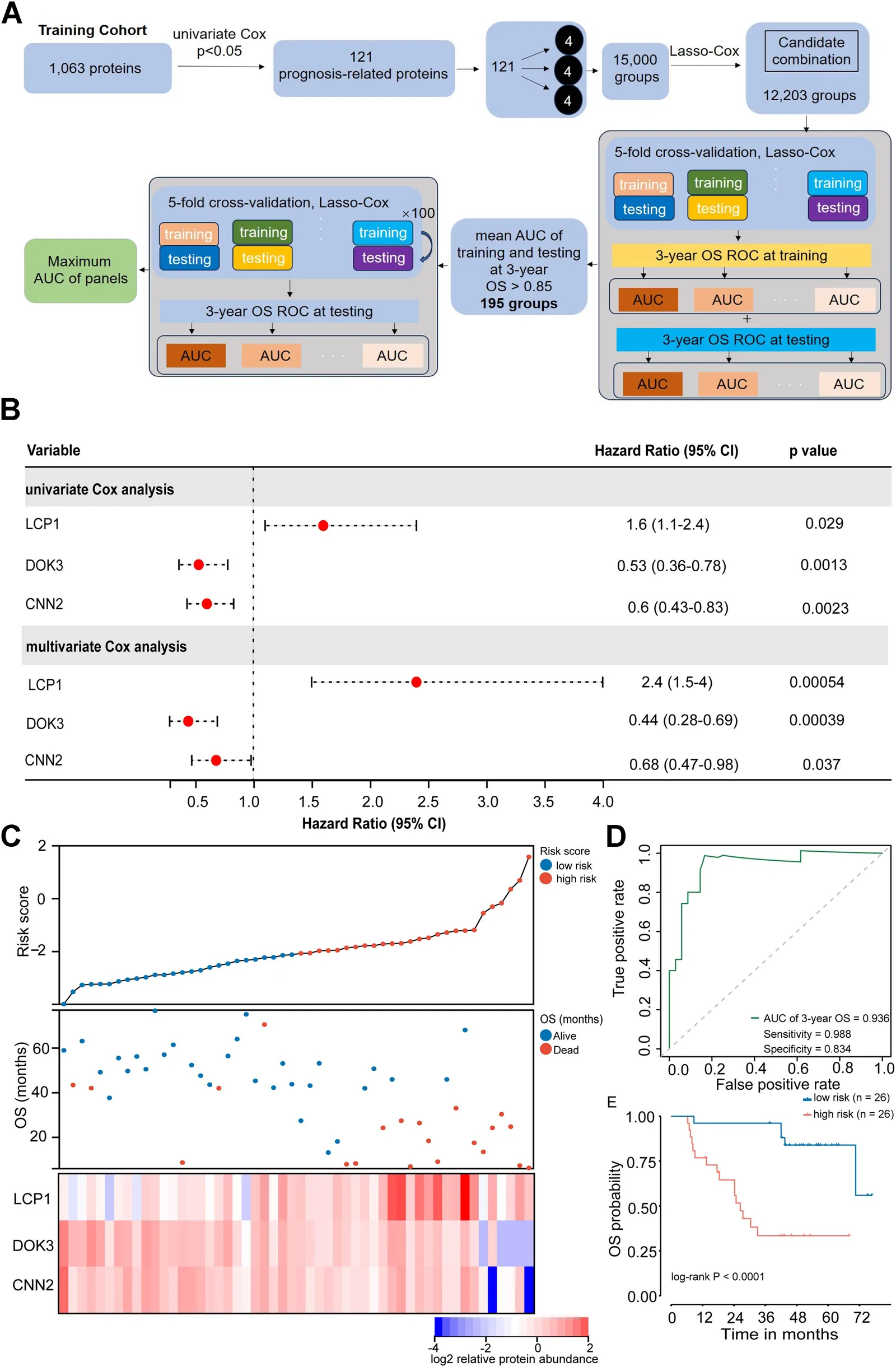
**Identification of potential biomarker combinations stratifying the prognostic risk of patients with BCLC stage B HCC in the training cohort by using a Lasso-Cox machine-learning strategy.***A*, the workflow of optimal prognostic marker panel screening. *B*, univariate and multivariate Cox regression analysis of three proteins in the training cohort. *C*, the distribution of risk score, survival status, and the expression of the three risk score proteins in the training cohort of the 52 samples (BCLC stages B HCC). In the first two pictures from *top to bottom*, *blue* represents low-risk (n = 26), while *red* denotes high-risk (n = 26) patients. The heatmap depicts the relative abundance of the three related proteins (log2 transformed). *D*, time-dependent ROC curves related to 3-year OS of the risk score in the training cohort. *E*, K-M curves of the OS between the low-risk and the high-risk groups predicted by median risk score for training cohort. BCLC, Barcelona Clinic Liver Cancer; Cox, Cox proportional hazards model; HCC, hepatocellular carcinoma; K-M, Kaplan-Meier; Lasso, Least Absolute Shrinkage and Selection Operator; OS, overall survival; ROC, receiver operating characteristic.

To evaluate the HRs of these proteins, univariate and multivariate Cox regression analyses were conducted (Fig. 3*B*). The coefficients of these three hub proteins in the panel revealed that LCP1 had an HR greater than 1 in both analyses, with a statistically significant *p*-value <0.05, identifying LCP1 being risky protein, and DOK3 and CNN2 exhibited HRs less than 1 (*p*-values <0.05), indicating they were protective proteins, respectively.

Based on the median value of risk score −0.047, we categorized training cohort of BCLC stage B HCC patients into two groups with different risk levels (Fig. 3*C*). The distribution of risk score values, OS status, and the expression profiles of the three risk score proteins for BCLC stage B HCC patients of the training cohort were illustrated in Figure 3*C*. The ROC curve demonstrated high accuracy of risk score with an AUC value of 0.936, a sensitivity of 0.988 and a specificity of 0.834 in predicting 3-years OS (Fig. 3*D*). K-M survival analysis showed that the patients in the low-risk group had better OS status (Fig. 3*E*), with the low-risk group having a mOS of 51.45 months compared to 24.52 months for the high-risk group (*p* < 0.0001), confirming the satisfactory predictive efficiency of this three-protein panel. Our prognostic risk model demonstrated that the low-risk group of BCLC stage B HCC had a favorable 3-years OS prognosis. Overall, these results support the model's ability to stratify HCC patients effectively, warranting further external validation.

### Estimation of the Prognostic Risk Score Model

Furthermore, both univariate and multivariate Cox regression analyses were conducted for OS in the training cohort, incorporating the prognostic risk score model alongside clinical characteristics, including gender, age, AFP levels, ALT, AST, TBIL, HBV DNA copy number, liver cirrhosis, and Kinki criteria. The analysis result of the risk score (HR 3.1, 95% CI 2-5; *p* < 0.001) confirmed that the combination of LCP1, CNN2, and DOK3 functions was as an independent prognostic factor for 3-year OS in patients with BCLC stage B HCC (Fig. 4*A*). Therefore, the prognostic risk score model we constructed can serve as both a standalone factor and a valuable tool for risk prediction, supporting surgical decision-making in BCLC stage B HCC patients.

**Fig. 4:**
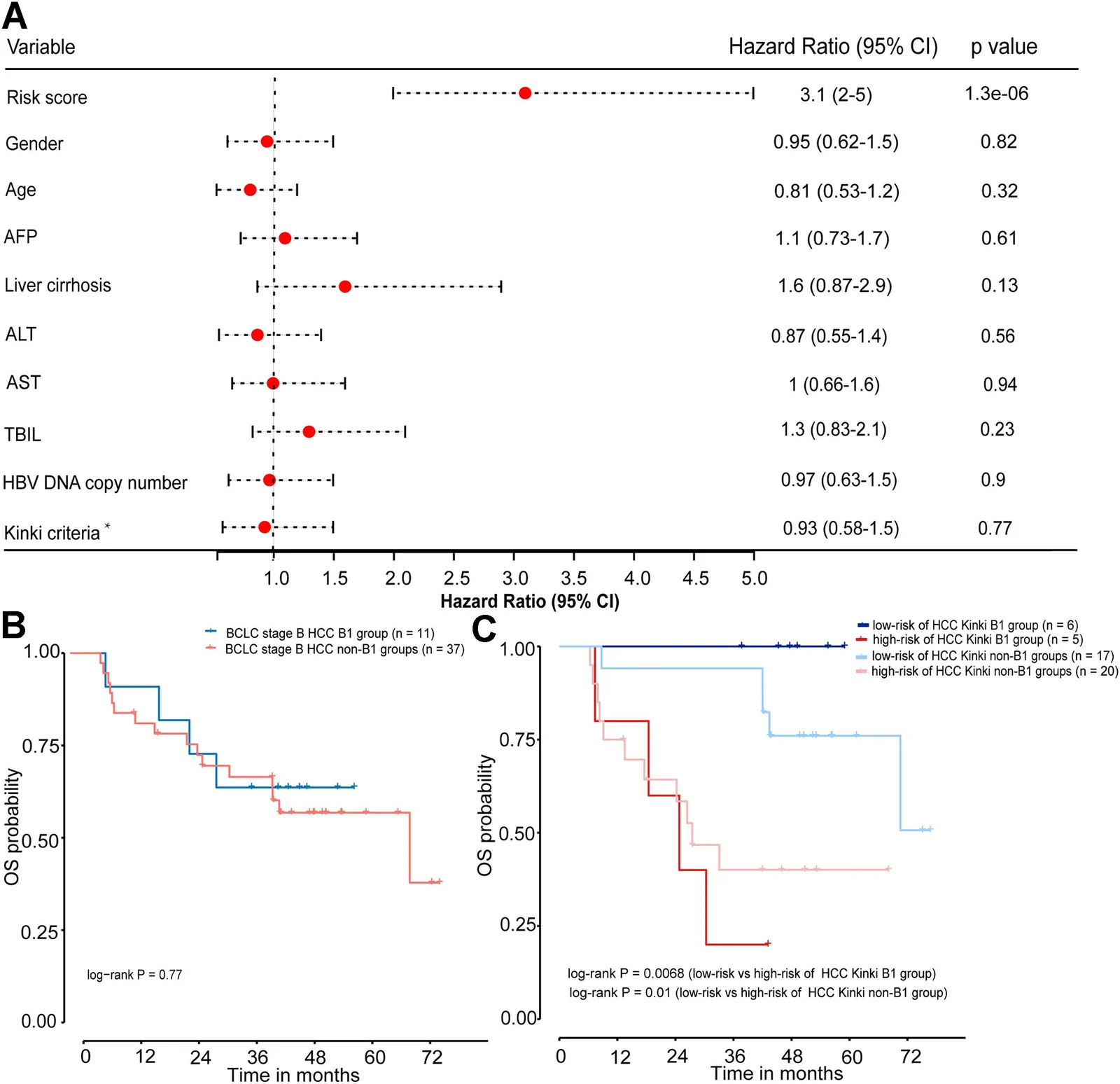
**Prognosis analysis according to the prognostic-risk score model in the training cohort.***A*, Univariable Cox regression analysis of risk score and other clinical baseline characteristics in the training cohort, indicating the independent factor of risk score in prognostic prediction. ∗ represents missing clinicopathological information for 4 patients. *B*, K-M curve of the OS in the training cohort stratified by the clinical Kinki criteria (N = 48, with clinicopathological information missing for 4 patients). The *p*-value from the log-rank test indicates poor performance in prognostic prediction. *C*, K-M curves of the OS between the low-risk and the high-risk groups predicted by the median risk score for the training cohort stratified by the clinical Kinki criteria. Cox, Cox proportional hazards model; K-M, Kaplan-Meier; OS, overall survival.

To evaluate the feasibility of our risk score, we compared our model with the clinical standard, the Kinki criteria. Based on this comparison, we categorized patients into two groups: B1-stage and non-B1-stage patients. Due to the lack of clinical information for 4 patients, a total of 48 patients with complete clinical data for the Kinki criteria were included in the analysis. Among these, 11 patients were classified in the B1 group (recommended for surgery) and 37 patients in the non-B1 group (not recommended for surgery). The K-M survival analysis using the Kinki criteria is presented in Figure 4*B*, with a log-rank test *p* value of 0.77, indicating no statistical significance.

Notably, when comparing our model with the clinical Kinki criteria, in the Kinki B1 group, our model classified 6 patients as the low-risk and 5 as the high-risk. In the Kinki non-B1 group, our model classified 17 patients as low-risk and 20 as high-risk. (Fig. 4*C* and Supplemental Table S9). The K-M survival analysis showed that the patients in the Kinki B1 low-risk group had better OS status, with the low-risk group having a median OS (mOS) of 49.05 months compared to 24.83 months for the high-risk group (*p* = 0.068) (Fig. 4*C*); the patients in the Kinki non-B1 low-risk group had better OS status (Fig. 4*C*), with the low-risk group having a mOS of 50.52 months compared to 26.94 months for the high-risk group (*p* = 0.01). Among the 17 patients classified as low-risk by our model, despite the fact that the Kinki criteria not recommending surgery, they experienced a favorable prognosis. This finding suggests that the prognostic risk model developed in our study provides better stratification of prognostic outcomes for BCLC stage B HCC patients following liver resection surgery, compared to the Kinki criteria. Therefore, our prognostic risk model demonstrated the low-risk group of BCLC stage B HCC had a favorable 3-years OS prognosis, confirming the robust prognostic value of this three-protein panel.

### The Performance of the Prognostic Risk Score Model in the Validation Cohort, the Extended Cohort and the Comparison Cohort

To validate the prognosis model, we used the validation, comparison, and extended cohorts with DIA identification. In the validation cohort of 22 patients with a mOS of 39.2 months, divided into low-risk (14 patients) and high-risk (8 patients) groups based on the median risk score, the distribution of risk scores, survival status, and protein expression profiles were consistent with the training cohort: LCP1 was up-regulated, while DOK3 and CNN2 were down-regulated (Fig. 5*A*). The 3-year AUC of 0.812, with a sensitivity of 0.875 and a specificity of 0.786, demonstrated strong predictive performance (Fig. 5*B*). K-M survival analyses revealed a significantly poorer mOS in the high-risk group compared to the low-risk group (low-risk mOS = 47.30 months; high-risk mOS = 22.07 months; *p* = 0.035) (Fig. 5*C*). Overall, our analyses demonstrated the robustness and accuracy of the prognosis model.

**Fig. 5:**
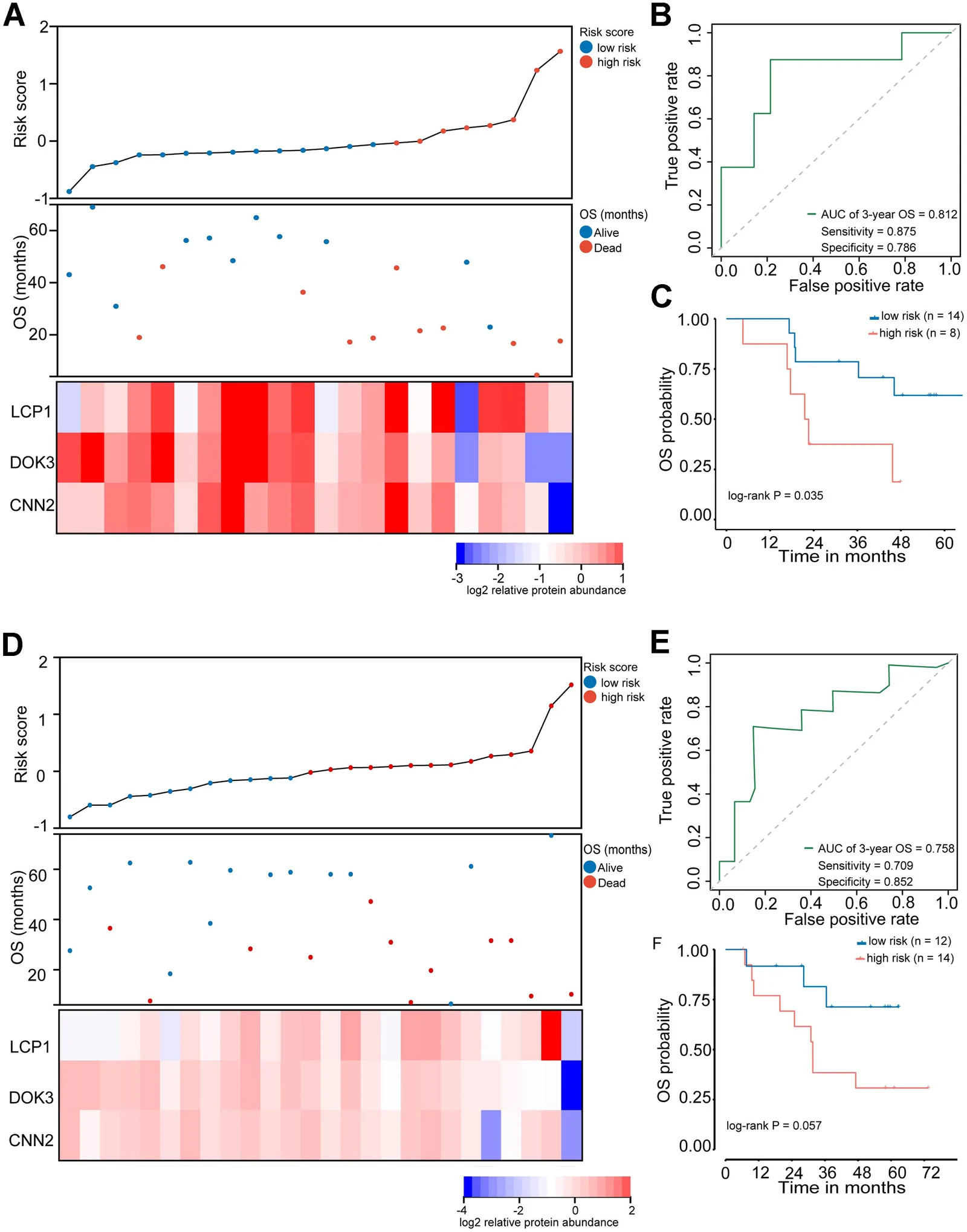
**The performance of prognosis risk model in the validation cohort and the extended cohort.***A*, the distribution of risk score, survival status, and the expression of the three risk score proteins in the validation cohort of the 22 patients with BCLC stage B HCC. In the first two pictures from *top to bottom*, *blue* represents low-risk (n = 14), while *red* denotes high-risk (n = 8) patients. The heatmap depicts the relative abundance of the three related proteins (log2 transformed). *B*, time-dependent ROC curves related to 3-years OS of the risk score in the validation cohort. *C*, K-M curves of the OS between the low-risk and the high-risk groups predicted by median risk score for the validation cohort. *D*, the distribution of risk score, survival status, and the expression of the three risk score proteins in the extended cohort of the 26 patients with a single huge HCC. In the first two pictures from top to bottom, blue represents low-risk (n = 12), while *red* denotes high-risk (n = 14) patients. The heatmap depicts the relative abundance of the three related proteins (log2 transformed). *E*, time-dependent ROC curves related to 3-years OS of the risk score in the extended cohort. *F*, K-M curves of the OS between the low-risk and the high-risk groups predicted by median risk score for the extended cohort. BCLC, Barcelona Clinic Liver Cancer; HCC, hepatocellular carcinoma; K-M, Kaplan-Meier; OS, overall survival; ROC, receiver operating characteristic.

Clinically, it is well-established that larger tumor diameters are associated with increased malignancy in patients with BCLC stage 0/A HCC, particularly in cases with huge HCC. Surgical decision-making for such patients remains challenging. In the extended cohort, we calculated risk scores for 26 patients (mOS = 37.7 months) with huge HCC and stratified them into low-risk (12 patients) and high-risk (14 patients) groups based on the median risk score. The distribution of the risk scores, survival status, and protein expression profile of the three risk score proteins were consistent with the training and validation cohort (Fig. 5*D*). The 3-years AUC of 0.758, a sensitivity of 0.709 and a specificity of 0.852 for OS prediction (Fig. 5*E*), K-M survival analyses also demonstrated a significantly poorer 3-years OS in the high-risk group compared with the low-risk group (low-risk mOS = 45.53 months; high-risk mOS = 31.27 months; *p* = 0.057) (Fig. 5*F*). These findings highlight that, by combining clinical characteristics with the prognostic risk score model, the prognosis model could possibly be applied to identify a subpopulation with a better prognosis among BCLC stage A HCC patients with huge HCC.

In the comparison cohort (N = 190, patients with BCLC stages 0/A HCC, excluding those with a single huge HCC), patients were also classified into low-risk (107 patients) and high-risk (83 patients) groups based on the same median value of risk score. The distribution of the risk scores, survival status, and protein expression profiles of the three risk score proteins are shown in Supplemental Fig. S5*A*. The 3-years AUC for predicting prognosis was 0.515 (Supplemental Fig. S5*B*). Unlike the training and validation cohorts, no clear trend in survival time was observed as risk score increased (Supplemental Fig. S5*C*). K-M analysis revealed no significant difference in 3-years OS between the low- and high-risk group (low-risk mOS = 52.60 months; high-risk mOS = 48.42 months; *p* = 0.83), indicating limited predictive value for BCLC stages 0/A patients except for those with huge HCC. Additionally, no significant prognostic difference was found between low-risk patients from BCLC stage B and those from the comparison cohort (Supplemental Fig. S6), or between low-risk patients from the extended cohort and those from the comparison cohort (Supplemental Fig. S7). These results provide further evidence that the low-risk BCLC stage B patients may benefit from surgery. The favorable protein signature likely mirrors less aggressive tumor biology, thereby supporting the feasibility of curative-intent resection.

### Orthogonal Validation of the Prognostic Risk Score Model

To orthogonally validate the prognostic risk score model, we performed PRM-based targeted quantification of the three-protein panel (LCP1, DOK3, and CNN2) in an independent cohort of 51 BCLC stage B serum samples. Following extensive empirical comparison of chromatographic behavior, ionization efficiency, and fragmentation patterns, the final surrogate peptides were selected as: EGIC[+57]AIGGTSEQSSVGTQHSYSEEEK (LCP1), LPELTRPQPC[+57]PLPR (DOK3), and SMQNWHQLENLSNFIK (CNN2), which consistently outperformed all other candidates, exhibiting the highest signal-to-noise ratios, cleanest MS/MS spectra with well-resolved b/y ion series, symmetric chromatographic peaks, and minimal interference from co-eluting matrix components across repeated injections (Supplemental Fig. S8 and Supplemental Table S10). Using the cutoff risk score generated from the DIA training cohort, patients were stratified into low-risk (n = 22) and high-risk (n = 29) groups. The PRM-derived protein expression levels were consistent with the training cohort (Fig. 6*A*). The 3-years AUC of 0.800, with a sensitivity of 0.870 and a specificity of 0.700 for OS prediction, confirmed robust predictive performance (Fig. 6*B*). The K-M survival analysis revealed significantly worse 3-years OS in the high-risk group compared with the low-risk group (low-risk mOS = 40.9 months; high-risk mOS = 23.6 months; *p* = 0.0025) (Fig. 6*C*). The orthogonal targeted proteome validation approaches substantiate the robustness, reproducibility, and generalizability of our prognostic risk score model.

**Fig. 6:**
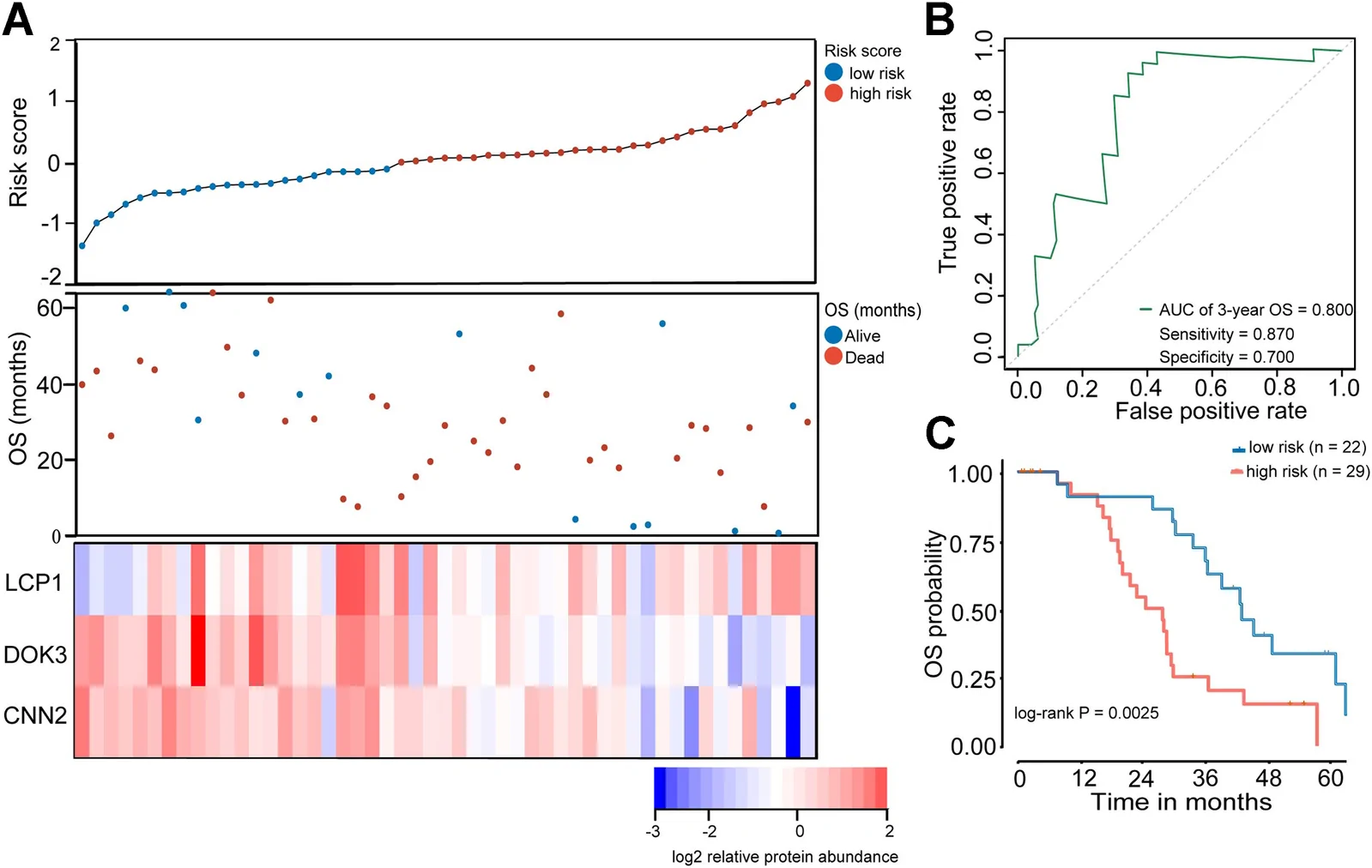
**Performance of the prognostic risk model in orthogonal validation of the exploration cohort using PRM proteome.***A*, distribution of risk score, survival status, and expression of the three peptides corresponding to three risk-score proteins in the exploration cohort of 51 patients with BCLC stage B HCC. In the first two panels (*top to bottom*), *blue* represents low-risk (n = 22), while *red* denotes high-risk (n = 29) patients. The heatmap depicts the relative abundance of the three related proteins (log2 transformed). *B*, time-dependent ROC curves for 3-year OS of the risk score in the exploration cohort with PRM proteome. *C*, K-M curves for OS between the low- and high-risk groups predicted by the risk score generated from the training cohort and applied to the exploration cohort. BCLC, Barcelona Clinic Liver Cancer; HCC, hepatocellular carcinoma; K-M, Kaplan-Meier; OS, overall survival; PRM, parallel reaction monitoring; ROC, receiver operating characteristic.

## Discussion

According to the most recent EASL and AASLD guidelines, patients with BCLC stage B HCC, particularly those with multinodular disease, are generally not candidates for curative treatments 31, 32. However, liver resection may still be considered in certain cases, even with more than four nodules (33). Given the heterogeneity of BCLC stage B HCC and the variability in treatment recommendations, the decision for surgery should be personalized. Therefore, an effective tool for surgical decision-making is essential. The Kinki criteria, which suggest that liver resection is more beneficial than TACE for B1 substage HCC with 5 to 7 tumors meeting the 'up-to-seven' criteria, provide clear guidance (13). However, these criteria require extensive assessments, including the Child-Pugh score and tumor burden evaluation. In contrast, proteomics offers high-throughput data that provides more comprehensive insights into a patient's condition. Recent advancements in integrating proteomic profiling with machine learning, specifically Lasso-Cox regression, show promise in survival analysis and early HCC recurrence prediction (34). Building on this, we applied a Lasso-Cox machine learning model combined with the serum proteome to identify and optimize biomarker panels for better stratification of BCLC stage B HCC patients before liver resection.

Recent systematic evaluations of nanoparticle-based plasma proteome have identified platelet and erythrocyte contamination as the major drivers of such variability, capable of artificially inflating protein identifications by thousands, even at low contamination levels 35, 36. Our observed range of 600–2300 identifications falls within the range reported in the literature for similar workflows (500–7000). However, high-depth serum proteome profiling has rarely been applied in disease-related cohort studies, largely due to the immense concentration range of serum proteins and the interference from highly abundant proteins. A promising solution involves enriching serum proteins using surface-functionalized nanoparticles, ensuring comprehensive coverage (18). Coupled with rigorous QC 27, 28 throughout the proteome analysis, this approach has proven effective and accurate for identifying early disease biomarkers. In our study, we employed the nanoparticle enrichment method, achieving the identification of 2378 proteins spanning six orders of magnitude in expression levels. The protein quantification correlation between QC samples exceeded 0.9 (Supplemental Fig. S1*A*), confirming the robustness of our methodology.

Integrating high-throughput proteome with AI algorithms (machine learning and deep learning) enabled the development of predictive models for novel biomarkers, enhancing BCLC stage B HCC stratification. We constructed a prognostic risk score model incorporating three protein markers (LCP1, DOK3, and CNN2) alongside clinical data, demonstrating strong performance in stratifying patients across training, validation, and extended cohorts. The model's predictive ability was validated using both Cox regression and comparisons with the Kinki criteria, showing superior discrimination between low- and high-risk patients. Notably, the model effectively predicted post-surgery outcomes, including a significant difference in survival between risk groups. Moreover, it outperformed the Kinki criteria in predicting outcomes for patients with a single huge HCC, underscoring its clinical applicability for both patients with BCLC stage B and patients with stage A HCC. The orthogonal validation of PRM-based targeted proteome not only confirmed the prognostic value of the three-protein panel of the risk score model at the protein level but also demonstrated that the observed serum expression patterns may mirror at the tissue level across independent external cohorts. The consistency between serum DIA and PRM data is particularly noteworthy, as it bridges the gap between circulating biomarker detection and tissue biology-a critical step toward clinical translation. The fact that all three proteins maintained their directional trends across four independent cohorts (training cohort, validation cohort, extended cohort, and PRM validation cohort substantially mitigates concerns regarding overfitting and cohort-specific bias, which are major pitfalls in multi-marker prognostic model development). Importantly, our validation in BCLC stage B patients-a heterogeneous group with controversial treatment recommendations-suggests that this model may assist in refining risk stratification and guiding therapeutic decisions, particularly in identifying patients who might benefit from more aggressive or earlier intervention.

In our prognostic risk score model, LCP1 is identified as a risk protein due to its HR value exceeding 1, whereas DOK3 and CNN2 are classified as protective proteins, as their HR values are less than 1. LCP1, an actin-binding protein initially isolated from human fibroblastoma (37), serves as a crucial regulator of cell migration and immune cell polarization 38, 39, 40. Numerous studies have documented its tumor-promoting effects in various cancer types, including gastric, oral, colorectal, and breast cancer 41, 42, 43, 44. Moreover, in human serum, the expression level of LCP1 has been significantly elevated in patients with simple fatty liver change, nonalcoholic steatohepatitis, and liver fibrosis compared to healthy individuals (45). On the other hand, DOK3, a member of the downstream tyrosine kinase family, facilitates IFN-β production *via* TRAF3/TBK1 complex formation and IRF3 activation 46, 47, 48. Research has highlighted its tumor-suppressing role in lung adenocarcinoma, with DOK3 knockout leading to the development of lung adenocarcinoma in mice (49). Conversely, Zhu *et al*. reported that DOK3 was a protective molecule in osteosarcoma (50). However, in the context of glioma, increased DOK3 expression is associated with suppressive immune cell infiltration and a poorer prognosis (49). CNN2, an actin cytoskeleton-binding protein that interacts with actin, calmodulin, and tropomyosin, has been implicated in cancer cell proliferation and metastasis 51, 52, 53. Specifically, it exhibits tumor-promoting effects in liver and colon cancers 54, 55. Together, previous studies emphasize the close association between these three proteins and cancer initiation, progression, and prognosis, indicating that alterations in their serum levels are intimately linked to disease status. To our knowledge, this is the first model capable of predicting the prognostic risk for BCLC stage B HCC patients prior to liver resection, thereby facilitating surgical decision-making.

Despite its promising performance, to further probe the limitations of our prognostic model, we analyzed individual misclassifications. While clinicopathological features (multiple tumors, TNM stages II-IIIA) did not differentiate false negative (FN) from false positive (FP) cases (Supplemental Table S11), their serum proteomic landscapes diverged sharply (Supplemental Fig. S9). FP cases (low-risk predicted as high-risk) paradoxically displayed a protective signature, marked by downregulation of pro-tumor mediators (DNAJB1, ICOSLG, ALDOC, CANX) 56, 57, 58, 59 and upregulation of tumor suppressors (RGS18, NBEAL2) 60, 61, suggesting that the model overweights generic stress signals while overlooking suppressor networks that actively restrain progression. In contrast, FN cases (high-risk predicted as low-risk) exhibited a covert aggressive profile, with coordinate upregulation of ECM-remodeling/lipogenic drivers (MMP2, TIMP1, LUM, FASN, C1QC) 61, 62, 63, 64 and silencing of homeostatic/redox regulators (GYPC) (65), indicating that the model underestimates signals of local invasion and metabolic reprogramming. These proteomic distinctions pinpoint actionable refinements: incorporating counter-balance modifiers for FP correction and upweighting rescue-target cascades for FN rescue should improve accuracy. Collectively, these findings affirm that misclassifications reflect biological heterogeneity and argue for integrating expression thresholds of these functional drivers in future model iterations to enhance clinical translation.

Nevertheless, the sample sizes in the training, validation, and PRM validation cohorts remain relatively limited. Therefore, the model requires further retrospective and prospective validation, and additional multi-centre cohorts would further enhance its robustness, reliability, and generalizability.

Moreover, owing to the absence of serum samples from patients with BCLC stage B HCC undergoing conservative treatment, the primary contribution of this study is currently confined to evaluating the prognosis of patients' post-surgery. Consequently, it is not feasible to provide a definitive overview of the patients' suitability for alternative treatments. Future endeavors should aim to gather more data to support and augment this work.

## Conclusion

In conclusion, the prognostic risk model, based on the high-depth serum proteomic profiling and a machine learning algorithm employing Lasso-Cox cross-validation integrating clinical characteristics, provides a strategy to stratify patients with BCLC stage B HCC. This model has the potential to predict prognostic risk and screen for low-risk patients when considering liver resection surgery, not only for BCLC stage B HCC patients but also for patients with a single huge HCC, thereby demonstrating the model's significant predictive and stratifying capabilities. The orthogonal validation of PRM-based targeted proteome also confirmed the feasibility and accuracy of the model. Integrating this model into clinical decision-making processes is anticipated to provide surgical suggestions that will mark a significant advancement in precision clinical decision-making, ultimately enhancing patients' treatment outcomes and quality of life through individualized treatment.

## Data Availability

Raw data of proteome derived from HCC serum has been deposited at iProX with the dataset identifier IPX0011253000 (accessible *via* https://www.iprox.cn/page/SSV024.html;url=1770691432812HuAp) with the accession number 6QeX and is publicly available as of the date of publication. All other data are available in the manuscript or the supplementary material.

## Code Availability

Data analysis was performed in R version 4.5.0. using custom or publicly-available R package. Individual packages are explicitly cited in the manuscript. The code is available upon request and deposited deposited at Zenodo: https://zenodo.org/records/18628379.

## Supplemental Data

This article contains supplemental data.

## Conflict of Interest

The authors declare that they have no conflicts of interest with the contents of this article.

## Figures and Tables

**Supplemental Fig. S1:**
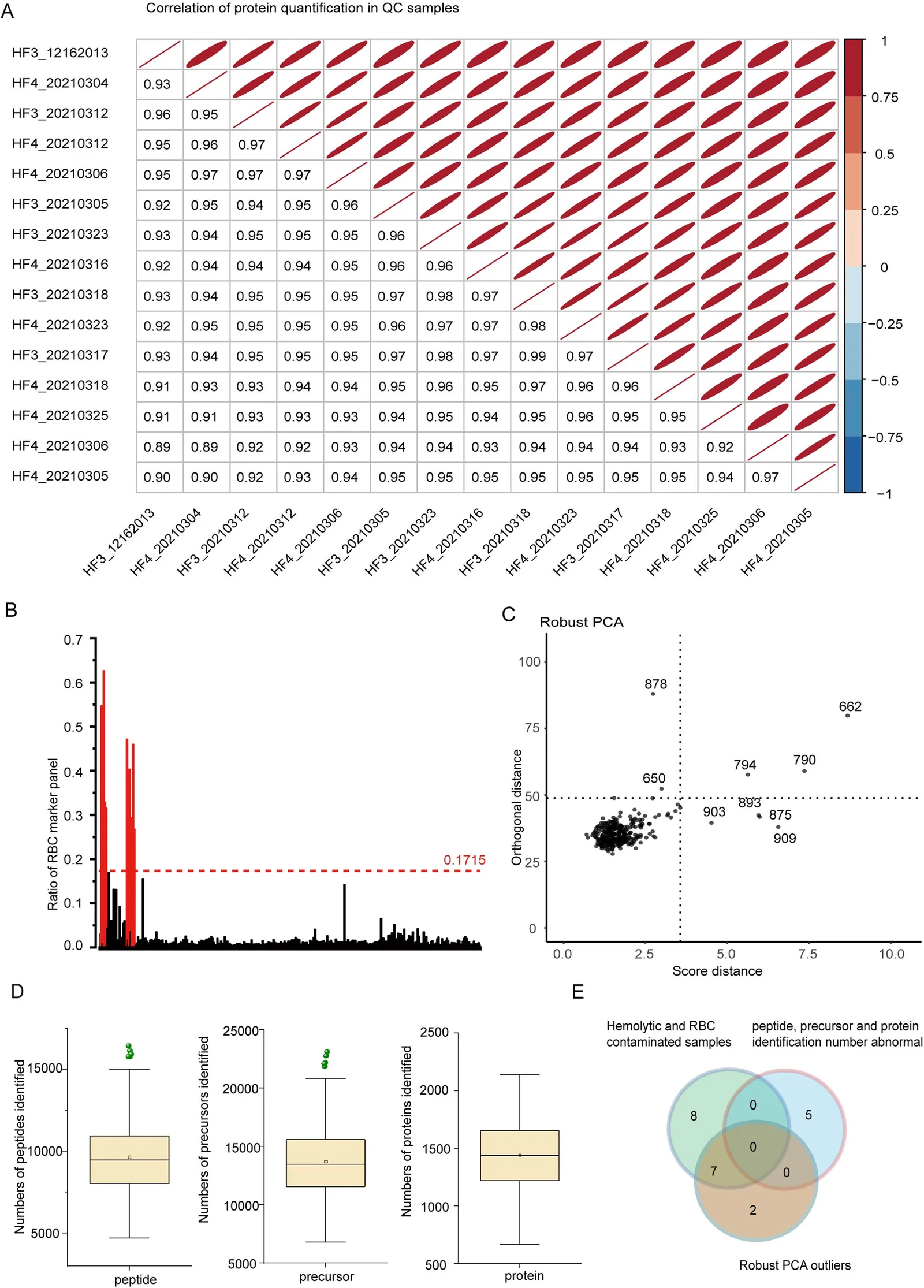
QC of serum sample proteome data. A, Pearson correlation analysis of protein quantification between quality control samples from different processing batches. B, Assessment of the percentage of erythrocyte contamination protein markers in the sample. The position of the red line is the mean plus two standard deviations, and samples exceeding this value are considered as contaminated samples. C, Robust PCA analysis of samples using all proteins. D, Box plot of peptide, precursor and identification protein number of 312 serum samples. E, Venn diagram of erythrocyte contaminated samples and robust PCA suggestive abnormal samples and identification number of protein, peptide, precursor of outlier samples. PCA, principal component analysis; QC, quality control.

**Supplemental Fig. S2:**
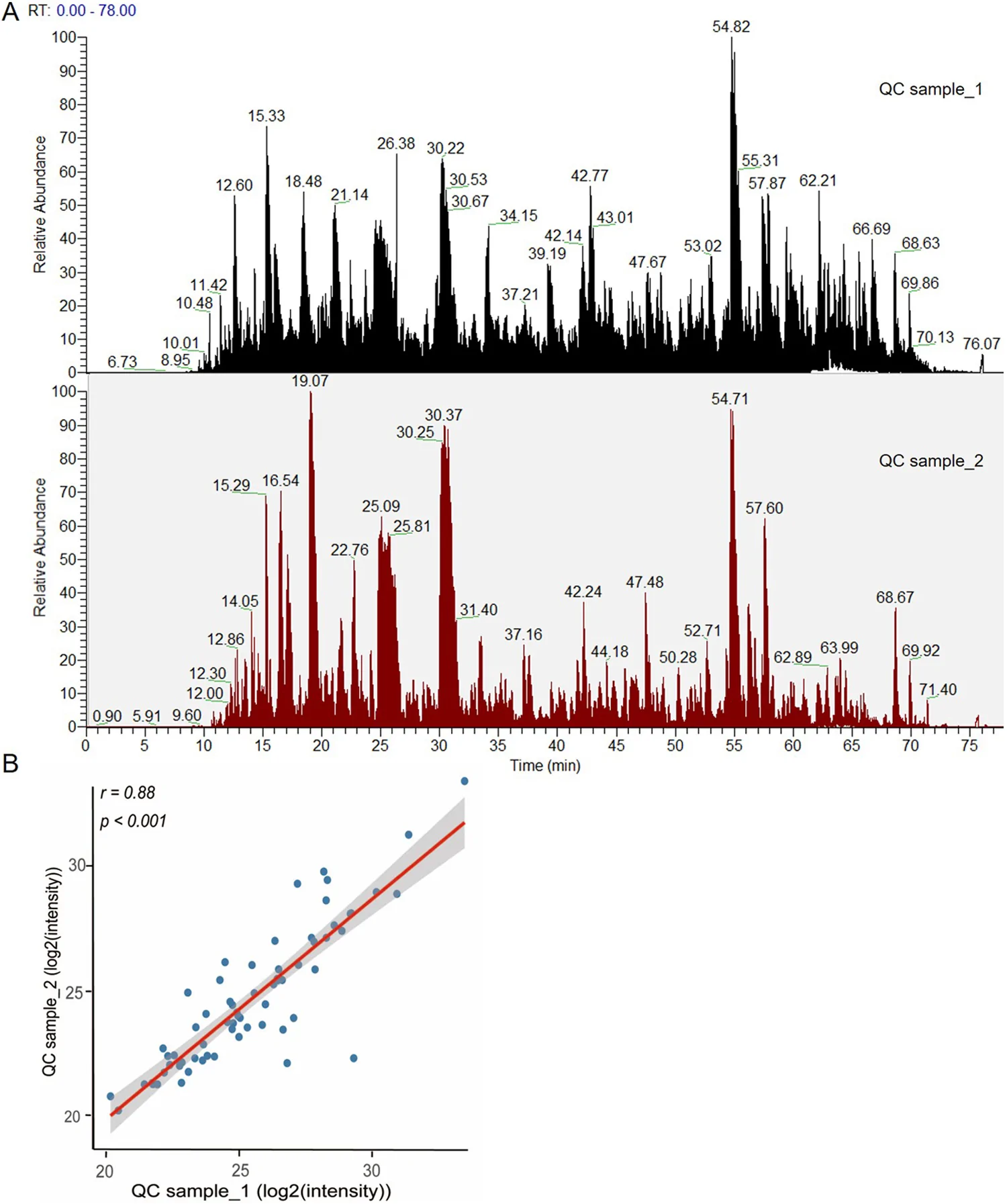
QC evaluation of PRM proteomic measurements. A, Base peak chromatograms (BPC) of QC samples. B, Linear regression scatter plot of log_2_-transformed protein intensities between QC samples. The solid red line represents the regression fit, gray shaded area indicates the 95% confidence interval. Pearson correlation coefficient (r) and P value are annotated. BPC, Base Peak Chromatogram; QC, Quality Control; PRM, parallel reaction monitoring.

**Supplemental Fig. S3:**
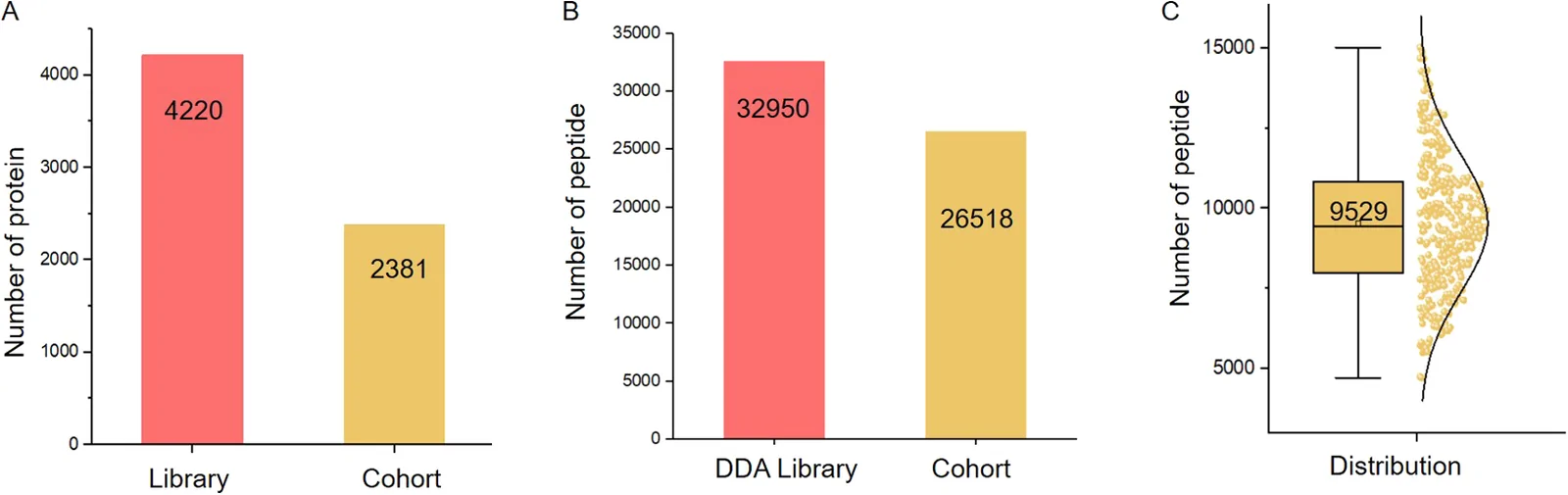
The output of MS-based serum proteomic profiling. A, The protein number identified in our DDA serum library and cohorts. B, The peptide number identified in our DDA serum library and cohorts. C, The distribution of peptide number in cohorts. DDA, data-dependent acquisition; MS, mass spectrometry.

**Supplemental Fig. S4:**
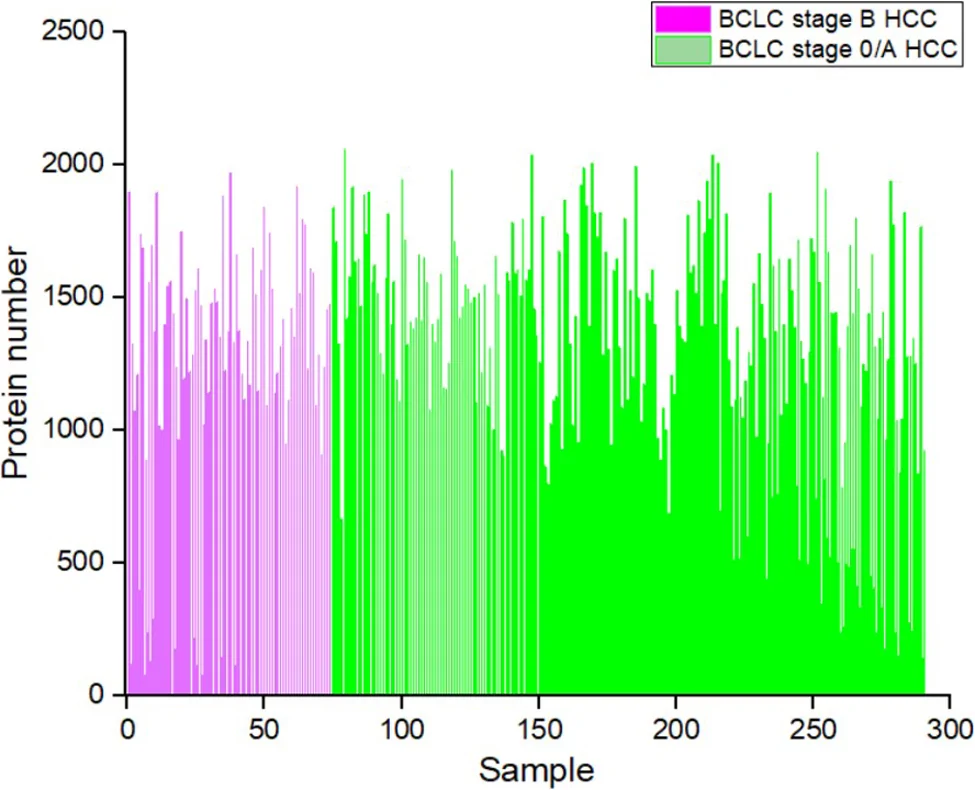
The number of proteins identified with DIA in each sample. DIA, data-independent acquisition.

**Supplemental Fig. S5:**
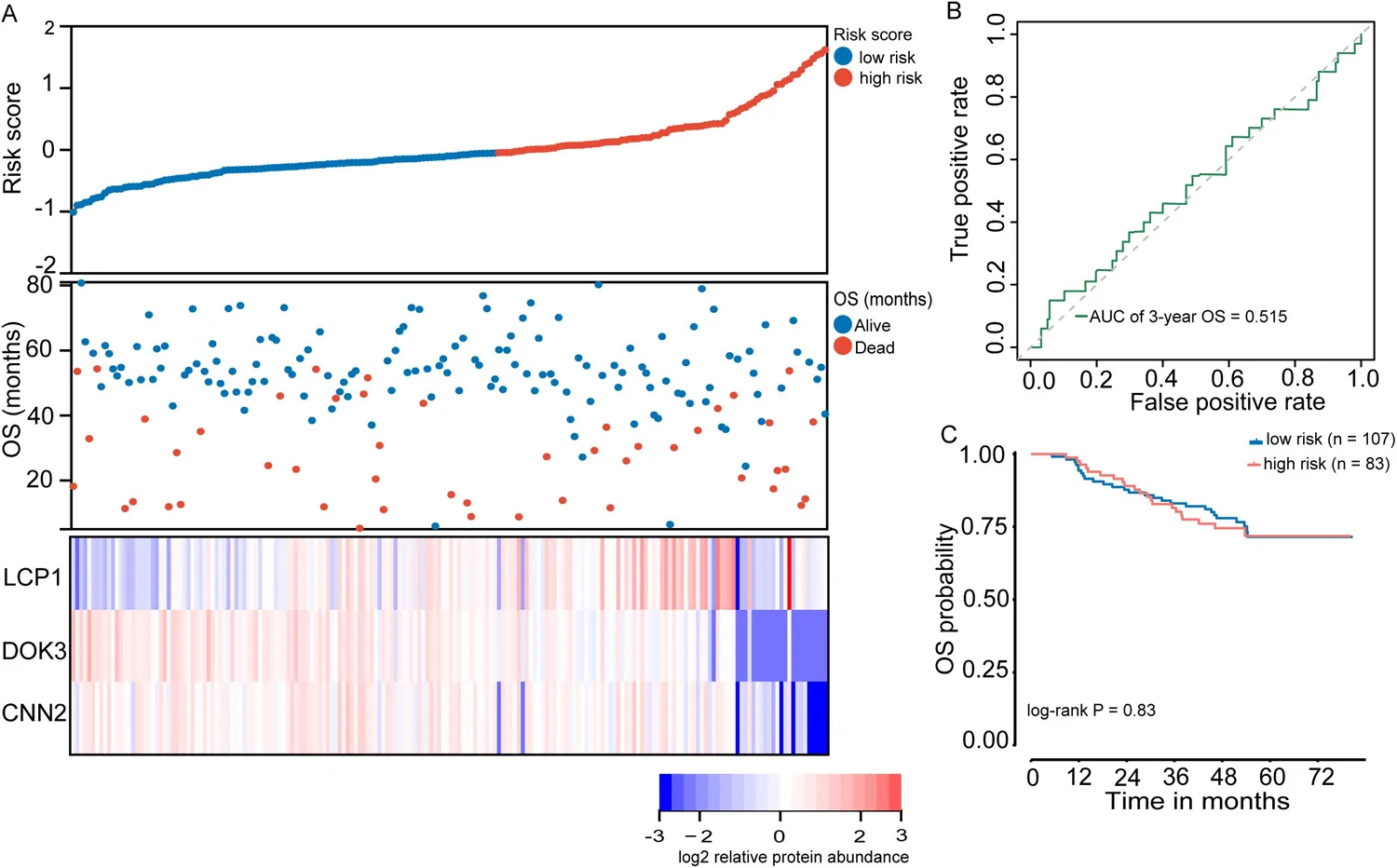
The performance of prognosis risk model in the comparison cohort. A, The distribution of risk score, survival status, and the expression of the three risk score proteins in the comparison cohort of the 190 BCLC stages 0/A HCC patients, except for those with huge HCC. In the first two pictures from top to bottom, blue represents low-risk (n = 107), while red denotes high-risk (n = 83) patients. The heatmap depicts the relative abundance of the three related proteins (log2 transformed). B, Time-dependent ROC curves related to 3-years OS of the risk score in the comparison cohort. C, K-M curves of the OS between the low-risk and the high-risk groups predicted by median risk score for the comparison cohort. BCLC, Barcelona Clinic Liver Cancer; HCC, hepatocellular carcinoma; K-M, Kaplan-Meier; OS, overall survival; ROC, receiver operating characteristic.

**Supplemental Fig. S6:**
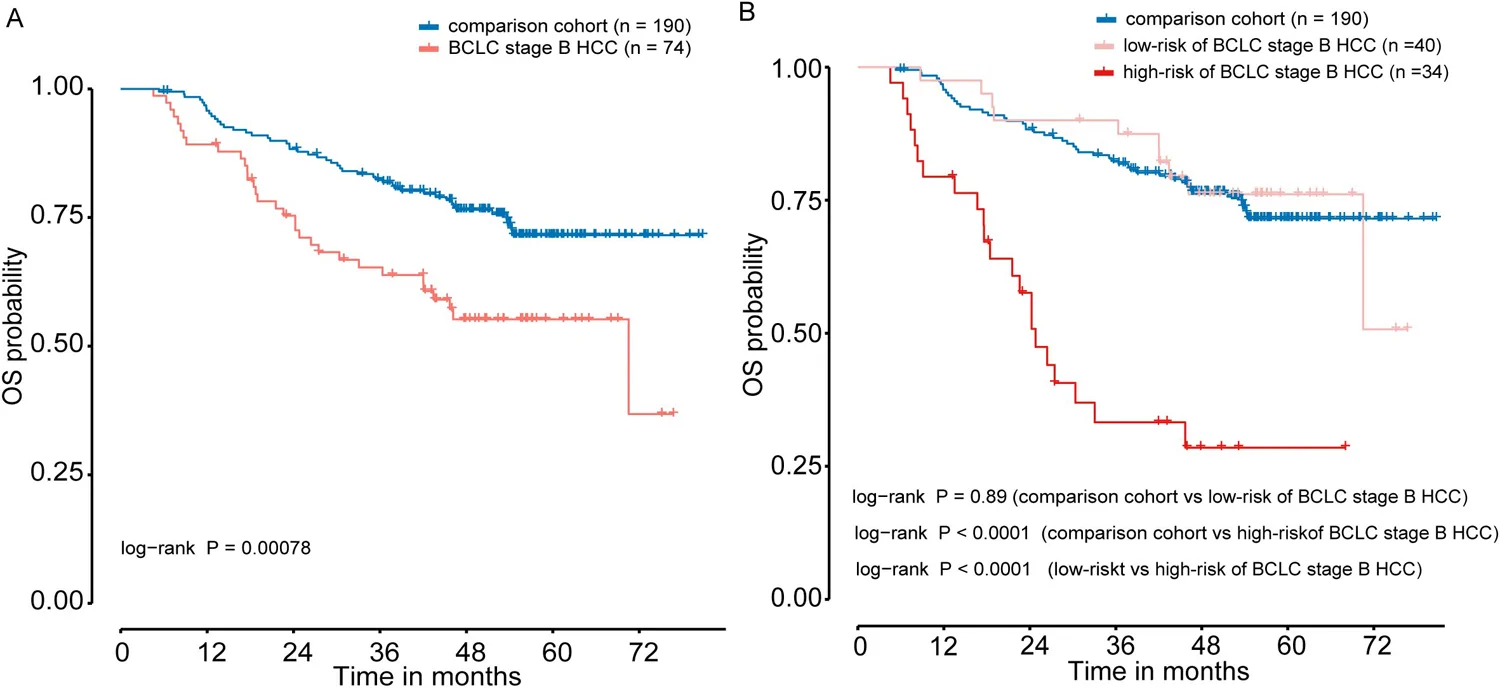
Comparison of K-M analysis for the BCLC stage B HCC patients and the comparison cohort. A, K-M curves of the OS between the comparison cohort of 190 BCLC stages 0/A HCC patients, except for those with huge HCC and 74 BCLC stage B HCC patients. B, K-M curves of the OS among BCLC stage B HCC patients grouping by median risk score and the comparison cohort, demonstrating patients classified as low-risk (n = 40) with significantly better 3-years OS compared to high-risk (n = 34) patients and no significant difference in prognosis between the comparison cohort and the low-risk of the BCLC stage B HCC patients. BCLC, Barcelona Clinic Liver Cancer; HCC, hepatocellular carcinoma; K-M, Kaplan-Meier; OS, overall survival.

**Supplemental Fig. S7:**
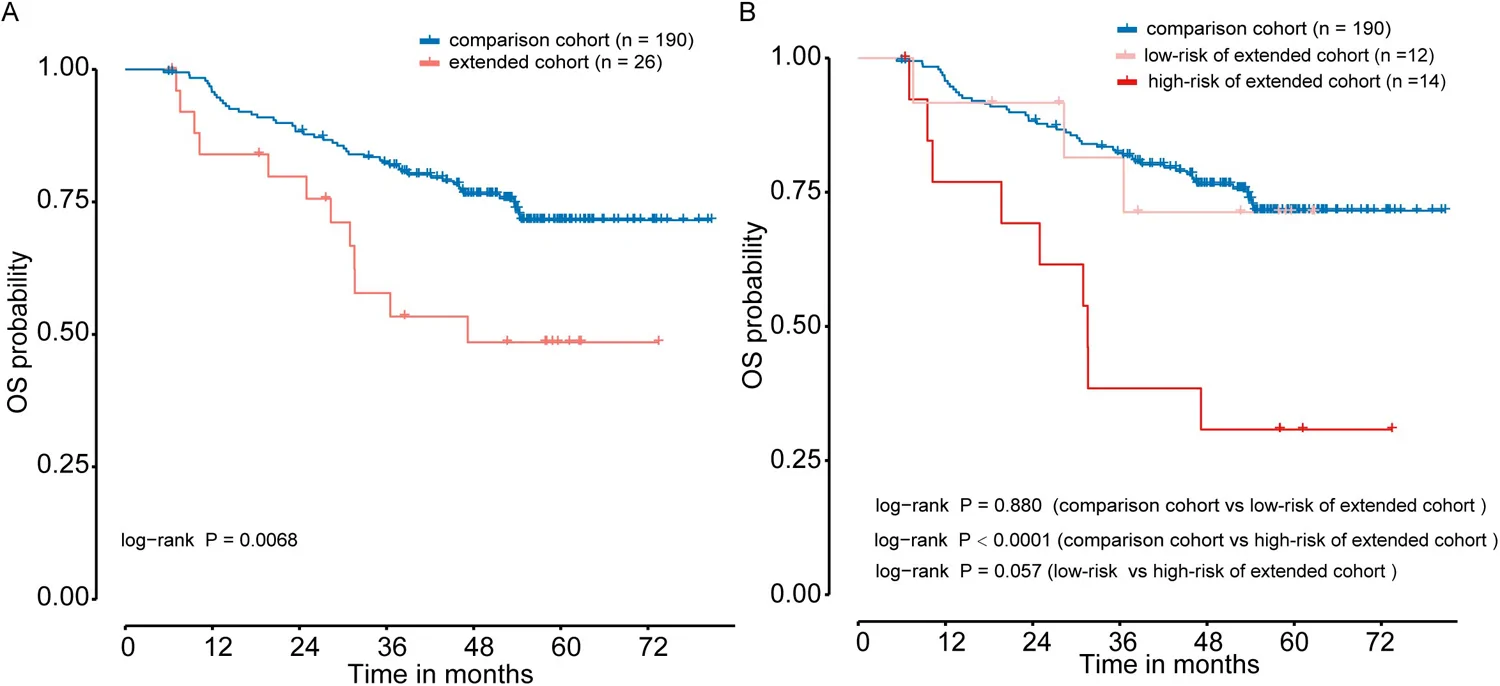
Comparison of K-M analysis for the extended cohort and the comparison cohort. A, K-M curves of the OS between the comparison cohort of 190 BCLC stages 0/A HCC patients, except for those with huge HCC and the extended cohort of 26 patients with a single huge HCC. B, K-M curves of the OS among the extended cohort grouping by median risk score and the comparison cohort, demonstrating patients classified as low-risk (n = 12) with significantly better 3-years OS compared to high-risk (n = 14) patients and no significant difference in prognosis between the comparison cohort and the low-risk patients of the extended cohort. BCLC, Barcelona Clinic Liver Cancer; HCC, hepatocellular carcinoma; K-M, Kaplan-Meier; OS, overall survival.

**Supplemental Fig. S8:**
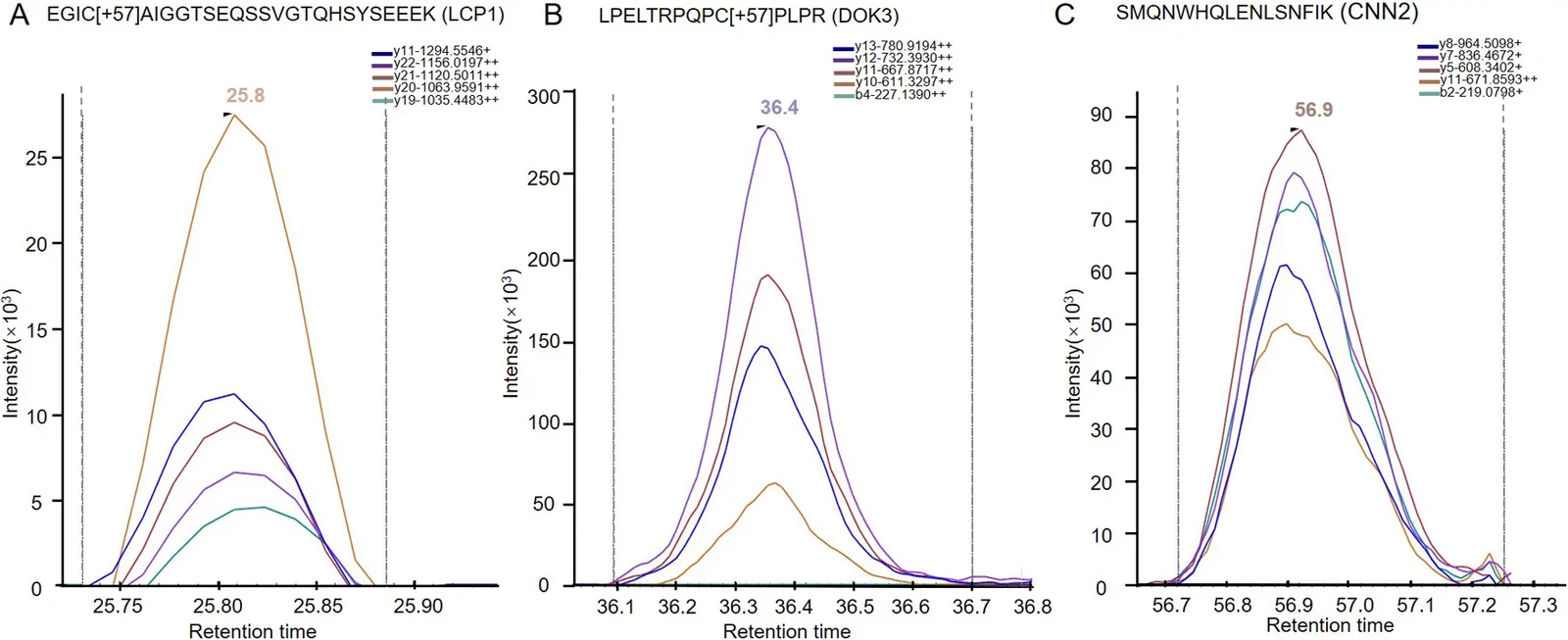
Orthogonal validation of three candidate proteins: representative PRM MS/MS transitions and fragment ion spectra used for quantitative analysis. A, EGIC[+57]AIGGTSEQSSVGTQHSYSEEEK for LCP1. B, LPELTRPQPC[+57]PLPR for DOK3. C, SMQNWHQLENLSNFIK for CNN2. MS/MS, tandem mass spectrometry; PRM, parallel reaction monitoring. [+57] denotes carbamidomethylation of cysteine.

**Supplemental Fig. S9:**
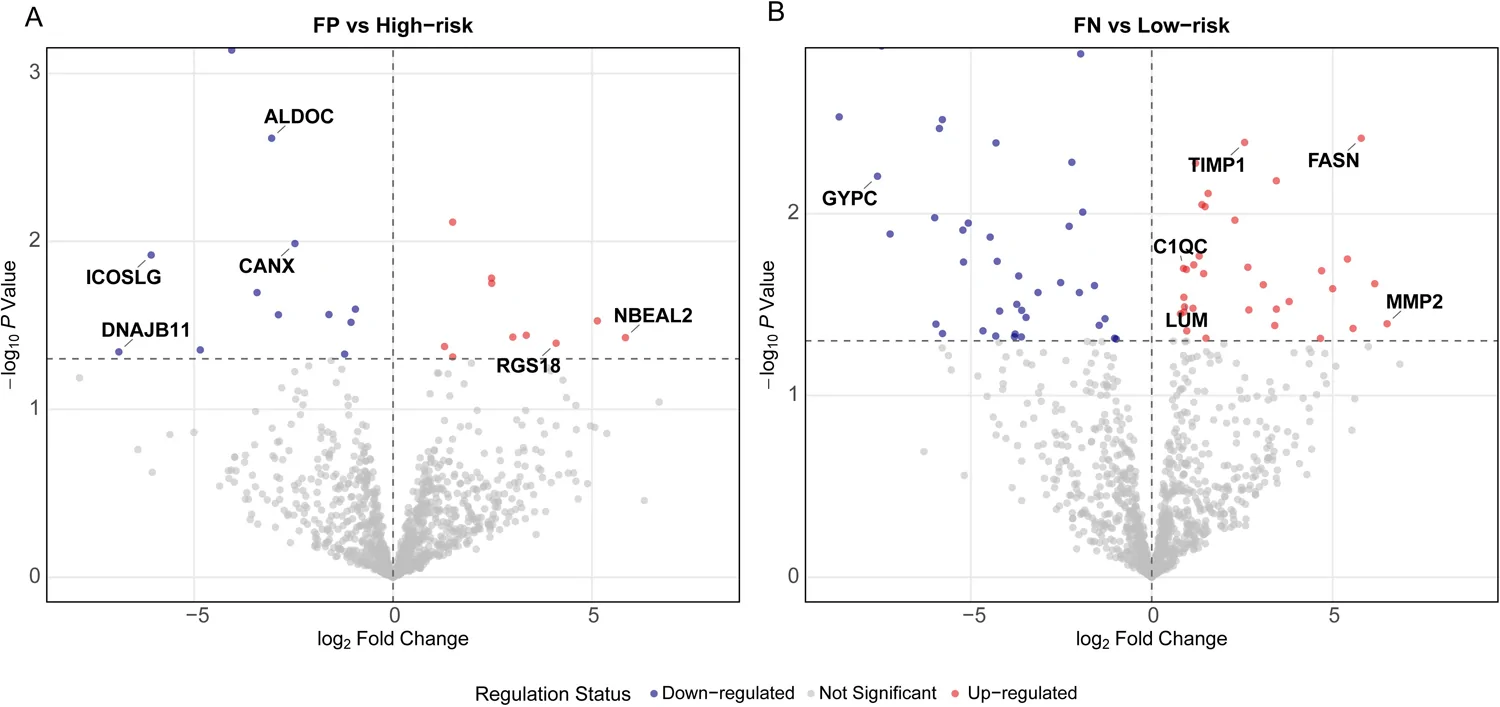
Volcano plots of differentially expressed proteins in misclassified patient cases. A, FP *versus* high-risk. B, FN *versus* low-risk. FN, false negative; FP, false positive.

## Supplemental data

Supplemental Table S1 Demographic and clinicopathological information of 290 HCC patients.

Supplemental Table S3 Demographic and clinicopathological features of the BCLC stage B HCC PRM validation cohort.

Supplemental Table S4 Peptides identified in DDA analysis for spectral library generation.

Supplemental Table S5 MS-based protein identification in 312 serum samples from HCC patients.

Supplemental Table S6 List of protein contaminants in QC filtration.

Supplemental Table S7 Peptide list identified by DDA analysis (MaxQuant) for PRM acquisition.

Supplemental Table S10 PRM-based quantification of target peptide fragment ion peak areas in the validation cohort (N = 51, BCLC stage B HCC).

Supplemental Table S11 Clinical features of misclassified patients in the validation cohort.
