# Supplementary material for: Serum Protein Panel Facilitates Surgical Decision-Making for Barcelona Stage B Hepatocellular Carcinoma
Source: Mol Cell Proteomics. 2026 Oct 5;25(11):101674. doi: 10.1016/j.mcpro.2026.101674 (PMC13743420; doi:10.1016/j.mcpro.2026.101674)
Supplement: Supplementary file 1 [file mmc9.docx]

Supplementary Information

**Serum Protein Panel Facilitates Surgical Decision-Making for Barcelona Clinic Liver Cancer Stage B Hepatocellular Carcinoma**

Jin-Hua Wang, Liang-Liang Ren, Wan-Jun Zhang, Xiao-Hua Xing, Jun-Qing Li, Chao-Ying Li, Wei-Jie Qin, Xiao-Long Liu, and Ying Jiang

**Synthesis of hydroxyl-modified SiO_2_ nanoparticles, surface amino-modified SiO_2_ nanoparticles, zwitterionic-modified SiO_2_ nanoparticles, and TiO_2_ nanoparticles**

The surface-hydroxyl-modified SiO₂ nanoparticles was synthesized rough a modified Stöber process as reported before (1, 2). Typically, 9 mL of 28% ammonia solution, 16.25 mL of ethanol, and 24.75 mL of deionized water are mixed. A 50-mL ethanol solution of Tetraethyl orthosilicate (TEOS) with a concentration of 9% is rapidly added to the mixture. After stirring for 1 minute, the stirring speed is reduced to 300 rpm, and the mixture is sealed with a rubber stopper. The reaction continues at room temperature for 2 hours. The resulting suspension is then centrifuged at 2000 RCF to collect the product.

The surface-aminated SiO₂ nanoparticles were synthesized via the hydrolysis of silane coupling agents. Specifically, 1 mL of (3-aminopropyl) triethoxysilane (APTES) was added to 50 mL of ethanol under continuous stirring at 1000 rpm for 10 minutes at room temperature. Subsequently, 50 mg of surface-hydroxyl-modified SiO₂ nanoparticles were introduced into the mixture, and the reaction was allowed to proceed under magnetic stirring at room temperature for 24 hours. Upon completion of the reaction, the SiO₂ nanoparticles were repeatedly centrifuged and washed with anhydrous ethanol to eliminate any unreacted APTES. Finally, the amine-functionalized SiO₂ nanoparticles were freeze-dried to obtain a powdered product.

The TiO₂ nanoparticles were synthesized through the published method via solvothermal reaction (3). First, an acidic solution of titanium tetrachloride (TiCl₄) with a concentration of 0.2 mol/L was prepared in an ice-water bath. This solution was then slowly added dropwise to 50 mL of a 0.5 mol/L ammonia solution under continuous magnetic stirring until the pH of the system reached 8.0. After complete addition, the mixture was further stirred magnetically for 30 minutes. The resulting suspension was centrifuged to collect the white precipitate, which was washed three times with 20 mL of deionized water. The washed precipitate was dispersed in 50 mL of anhydrous ethanol via ultrasonication and transferred into a 100 mL polytetrafluoroethylene (PTFE)-lined stainless steel autoclave. The autoclave was placed in a forced-air oven and heated at 150°C for 10 hours. After the reaction, the obtained white solid product was ground and sieved through a 325-mesh screen to yield TiO₂ nanoparticles with a particle size distribution ranging from 200 to 500 nm.

The surface-zwitterionic-modified SiO₂ nanoparticles were synthesized through the following procedure: Initially, 50 mg of surface-hydroxyl-modified SiO₂ nanoparticles were washed three times with methanol. Subsequently, 200 µL of a 2 mM solution of 2-bromo-2-methyl-N-(3-(triethoxysilyl)propyl) propionamide (SI-ATRP initiator) was added to the nanoparticles. The reaction mixture was maintained at 37°C for 12 hours. Following the reaction, the nanoparticles were subjected to centrifugation to remove the supernatant and washed three times with 500 µL of methanol. The SI-ATRP polymerization solution, composed of N, N-dimethyl-N-(acryloyloxyethyl)-N-(3-sulfopropyl) ammonium chloride, copper (I) chloride, copper (II) chloride, and 1,1,4,7,7-pentamethyldiethylenetriamine in a molar ratio of 200:1:0.1:1.5, was prepared by dissolving the components in 700 µL of anhydrous toluene and vortexing to ensure homogeneity. This polymerization solution was then added to the treated SiO₂ nanoparticles. The mixture was sealed and allowed to react for 4 hours. After reaction, the nanoparticles were washed three times with 500 µL of anhydrous toluene. The final product, a white powder, was obtained after vacuum drying and exhibited a particle size distribution ranging from 200 to 500 nm. This powder represents the surface-zwitterionic-modified SiO₂ nanoparticles.

**Table S2** Clinical baseline features of the extended and comparison cohorts

|  | BCLC stage 0/A HCC Patients | | |
| --- | --- | --- | --- |
|  | Total | Extended cohort  Single tumor, diameter ≥ 10 cm | Comparison cohort  Tumor diameter ＜10 cm |
| Variable | （ N = 216） | （ N = 26 ） | （ N = 190） |
| Baseline information |  |  |  |
| Gender |  |  |  |
| Female | 27 | 2 | 25 |
| Male | 189 | 24 | 165 |
| Age, year |  |  |  |
| ≤ 60 | 143 | 20 | 123 |
| ＞ 60 | 73 | 6 | 67 |
| Laboratory test |  |  |  |
| AFP ( ng/ml ) |  |  |  |
| ≤ 400 | 174 | 14 | 160 |
| ＞400 | 42 | 12 | 30 |
| ALT (u/l) |  |  |  |
| < 50 | 157 | 13 | 144 |
| ≥ 50 | 59 | 13 | 46 |
| AST (u/l) |  |  |  |
| < 40 | 146 | 7 | 139 |
| ≥ 40 | 70 | 19 | 51 |
| TBIL (μl/mol) |  |  |  |
| < 17.1 | 212 | 14 | 98 |
| ≥ 17.1 | 104 | 12 | 92 |
| HBV DNA copy number |  |  |  |
| < 2000 | 136 | 15 | 121 |
| ≥2000 | 80 | 11 | 69 |
| Liver function |  |  |  |
| Liver cirrhosis |  |  |  |
| absence | 47 | 13 | 34 |
| present | 169 | 13 | 156 |
| Tumor characteristics |  |  |  |
| TNM stage |  |  |  |
| II | 80 | 14 | 66 |
| IIIA | 138 | 12 | 124 |
| Diameter of tumor(cm) |  |  |  |
| ≤ 10 | 194 | 4 | 190 |
| ＞ 10 | 22 | 22 | 0 |
| Tumor Number |  |  |  |
| 2 | 9 | 0 | 9 |
| ≥ 3 | 0 | 0 | 0 |
| Microvascular Invasion |  |  |  |
| 0 | 142 | 12 | 130 |
| 1 | 74 | 14 | 60 |
| Differentiation |  |  |  |
| Edmondson I-II | 74 | 8 | 66 |
| Edmondson III-IV | 142 | 18 | 124 |

Note: BCLC, Barcelona Clinic Liver Cancer; HCC, Hepatocellular carcinoma; AFP, Alpha‑fetoprotein; ALT, Alanine transaminase; AST, Aspartate transaminase; TBIL, Total bilirubin; HBV‑DNA, Hepatitis B virus‑DNA; TNM, Tumor‑Node‑Metastasis; MVI, Microvascular invasion; Edmondson‑Steiner, Edmondson‑Steiner grading system.

**Table S8** Key resource table (STAR method)

| **Chemicals, peptides, and recombinant proteins** | | |
| --- | --- | --- |
| 1M Tris-HCI, pH 8.0 | Thermo Fisher | Cat # 15568-025 |
| Sequencing Grade Modified Trypsin | Progema | Cat # V5111 |
| 3-((3-Cholamidopropyl) dimethylamm  -onio)-1-propanesulfonate (CHAPS) | Sigma Aldrich | Cat # 220201 |
| Sodium deoxycholate (SDC) | Sigma Aldrich | Cat # [264103](https://www.sigmaaldrich.cn/CN/zh/product/mm/264103) |
| Tris(2-carboxyethyl) phosphine hydro  -chloride (TCEP) | Sigma Aldrich | Cat # [646547](https://www.sigmaaldrich.cn/CN/zh/product/sigma/646547) |
| Chloroacetamide (CAA) | Sigma Aldrich | Cat # [C0267](https://www.sigmaaldrich.cn/CN/zh/product/aldrich/c0267) |
| 1M Triethylammonium Bicarbonate  (TEAB) | Thermo Fisher | Cat # 90114 |
| Trifluoroacetic acid (TFA) | Sigma Aldrich | Cat # T6508 |
| Tetraethyl orthosilicate (TEOS) | Sigma Aldrich | Cat # 86578 |
| Ammonium hydroxide | Sigma Aldrich | Cat # 221228 |
| Ethanol | Beijing Chemical Reagent Company | Cat # 117902162 |
| (3-Aminopropyl) triethoxysilane | Sigma Aldrich | Cat # [A3648](https://www.sigmaaldrich.cn/CN/zh/product/sial/a3648) |
| Titanium(IV) chloride | Sigma Aldrich | Cat # 8123821000 |
| Copper(I) chloride | Sigma Aldrich | Cat # 212946 |
| Copper(II) chloride | Sigma Aldrich | Cat # 751944 |
| [2-(Methacryloyloxy) ethyl]dimethyl-(3-sulfopropyl) ammonium hydroxide | Sigma Aldrich | Cat # 537284 |
| Acetonitrile | Sigma-Aldrich | Cat # 766445-100MG |
| iRT | Biognosys | Cat # KI-3002-2 |
| **Software and algorithms** |  |  |
| R (version 4.1.3) | R Core Team | https://www.r-project.org |
| Spectronaut (version14.10.201222) | Biognosys Inc. | https://www.biognosys.com |
| UniProt Human database (version 2021. 11.05) | UniProtKB | <https://www.uniprot.org/>  RRID:SCR_004426 |
| Maxquant (version 2.0.3.0) | Max-Planck-Institute | https://www.maxquant.org/  RRID:SCR_014485 |
| Perseus (version 1.6.14.0) | Max-Planck-Institute | http://www.perseus-framework.org  RRID:SCR_015753 |
| Sangerbox (version 3.0) | Mugu, Hangzhou | http://www.sangerbox.com/home.html |

**Table S9** The orthogonal array number of the training cohort of 48 patients classified by the prognostic risk model and Kinki criteria

|  | **Low risk** | **High risk** | **Total** |
| --- | --- | --- | --- |
| **Kinki_B1** | 6 | 5 | 11 |
| **Kinki_non-B1** | 17 | 20 | 37 |
| **Total** | 23 | 25 | 48 |

**Reference**

1. Noreen, S., Maqbool, A., Maqbool, I., Shafique, A., Khan, M., Junejo, Y. M., Ahmed, B., Anwar, M., Majeed, A., Abbas, M. M. (**2022)**. Multifunctional mesoporous silica-based nanocomposites: synthesis and biomedical applications. *Materials Chemistry and Physics*, **285**, 126132.

2. Teng, Z., Sun, C., Su, X., Liu, Y., Tang, Y., Zhao, Y., Chen, G., Yan, F., Yang, N., Wang, C., & Lu, G., (2013). Superparamagnetic high-magnetization composite spheres with highly aminated ordered mesoporous silica shell for biomedical applications. *Journal of Materials Chemistry. B*, **1**(36), 4684-4691.

3. Liu, J., Sun, Z., Deng, Y., Zou, Y., Li, C., Guo, X., Xiong, L., Gao, Y., Li, F., & Zhao, D. (2009). Highly water-dispersible biocompatible magnetite particles with low cytotoxicity stabilized by citrate groups. *Angewandte Chemie (International ed. in English)*, **48**(32), 5875-5879.
